# The Hitchhiker’s Guide to the Surface Code

**DOI:** 10.3390/e28020251

**Published:** 2026-02-22

**Authors:** Fang Zhang, Jianxin Chen

**Affiliations:** 1 Zhongguancun Laboratory, Beijing 100095, China; 2 Department of Computer Science and Technology, Tsinghua University, Beijing 100084, China; chenjianxin@tsinghua.edu.cn

**Keywords:** quantum error correction, surface code, lattice surgery

## Abstract

Error correction is an essential part of the theory of quantum computation. However, new quantum computation students may find the theories of error correction and fault tolerance daunting, or they may be stuck with theoretical/outdated schemes (such as the one in the original proof of the threshold theorem by Aharonov and Ben-or) with unrealistically low thresholds and/or high overhead. In this article, we describe an adequately modern approach to fault-tolerant quantum computation based on the surface code and lattice surgery. The reader is assumed to have a basic understanding of quantum computation (state vectors, unitary gates, and measurements, etc.), but no prior knowledge about quantum codes or quantum error correction is needed.

## 1. An Overview of Quantum Error Correction

Error correction codes are a familiar concept in information science, even in classical computation and even in places where one may not think error correction is necessary at all. For example, the electrical current in any tiny segment of copper wire is encoded in the movements of at least trillions of free electrons. This can be roughly regarded as a *repetition code*: The information is repeated trillions of times so that even when some electrons are moving in the “wrong” direction—they *will* do so, due to microscopic fluctuation—the correct macroscopic information will be preserved and processed by the overall circuit.

Quantum computation is essentially no different, except that handling quantum information is a rather more subtle task. The no-cloning theorem [1] states that it is impossible to make a perfect independent clone of a qubit (unless we have some specific a priori knowledge of the state of the qubit, e.g., if we know that it is either |0〉 or |1〉 ). In fact, a form of the complementarity principle implies that any physical procedure that can perfectly distinguish between |0〉 and |1〉 must completely destroy any other information stored in the qubit, such as the distinction between |+〉 and |−〉 , and vice versa. The no-cloning theorem follows from this principle because cloning a qubit and then measuring the clone would have violated the no-cloning theorem.

Surprisingly, even under these restrictions, “protecting quantum information by making copies” is not complete nonsense. The trick is to think of two different types of information, the “*Z* information” ( |0〉 vs. |1〉 ) and the “*X* information” ( |+〉 vs. |−〉 ), and try to copy one type at a time. Suppose that, given a qubit, we want to make many copies of its *Z* information. In other words, we want to make many individual qubits (Really, we would have been satisfied with quantum systems with any Hilbert space dimension, but for this purpose it turns out that qubits suffice.) such that, whenever we want to, we can perfectly tell whether the original state was |0〉 or |1〉 by measuring *any* of those qubits. The complementarity principle tells us that, whenever that happens, the original *X* information must be completely destroyed. But we do not need to destroy it *now*, and it remains possible that as long as we never extract the *Z* information, we would have a way to extract the *X* information instead. The mandatory catch is that we would need to keep track of *every* copy to extract the *X* information. If we lose even one copy, then it becomes possible that an adversary would extract the *Z* information from it, and then *X* information extraction would become impossible. And the no-signaling principle means that it does not matter whether such an adversary actually exists.

To summarize, we want to find a way to encode one qubit into *n* qubits, such that:

- The *Z* information can be extracted from *any* one of those *n* qubits.
  - If at least 3 qubits are available, one could do a majority vote to defend against an error on an unknown qubit.
- The *X* information can be extracted from *all* of those *n* qubits.

Readers very familiar with the basics of quantum computation might find these requirements almost trivial to satisfy; hopefully, even if one does not immediately see an implementation, one should at least find it plausible that these requirements can be satisfied simultaneously. The construction is known as a *Z*-basis *quantum repetition code*, and the details will be left to Section 2.

Side note: Under the many-worlds interpretation of quantum mechanics [2], measuring a qubit essentially means encoding it into a repetition code with too many qubits. After a *Z*-basis measurement, the qubit becomes entangled with the entire macroscopic measuring apparatus and probably even with the whole environment around it. Even if it is in theory possible to undo the entanglement and recover the original *X* information, it would be impossible in practice to keep track of every microscopic particle involved in this entanglement. This is why, for practical purposes, quantum measurements do destroy complementary information.

**Figure 1:**
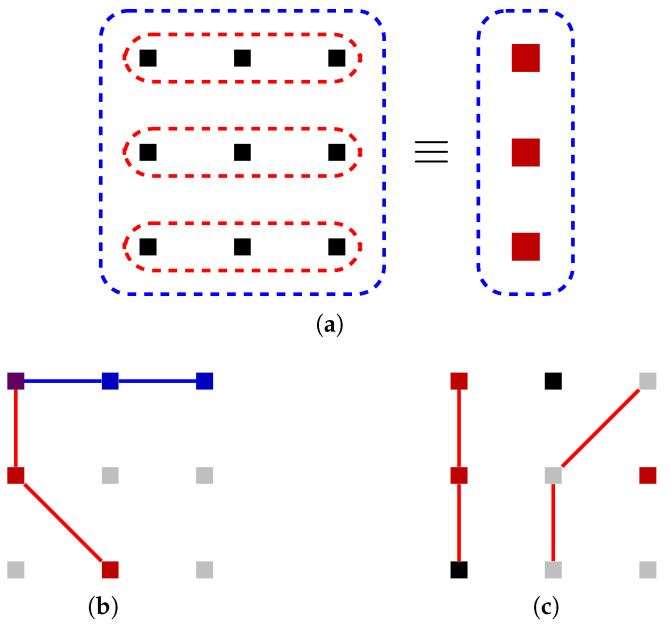
(**a**) The qubit arrangement of the 9-qubit Shor code. Each row is a 3-qubit *X*-basis repetition code (inner code), and all three rows form a 3-qubit *Z*-basis repetition code (outer code). (**b**) The original *Z* information can be extracted from the three qubits on the blue line, and the original *X* information can be extracted from the three qubits on the red line. Therefore, the logical qubit can be preserved even when 4 qubits are lost. However, note that this example depends on the exact locations of these 4 qubits. (**c**) If the locations of lost qubits are reflected by the main diagonal, then the original *Z* information is no longer recoverable because no three qubits in the same row remain. In fact, an adversary with the lost 4 qubits could *also* extract the *X* information, ruling out recovering the *Z* information from the remaining 5 qubits by the complementarity principle.

Of course, we still want a code that can protect both the *Z* information and the *X* information, not one at the cost of the other. But the quantum repetition code comes surprisingly close to achieving this goal. Consider this example: We encode a qubit in a 3-qubit *Z*-basis repetition code (the *outer code*), and then further encode each of the resulting 3 qubits in a 3-qubit *X*-basis repetition code (the *inner code*). The resulting 9 qubits can be arranged in a 3×3 rectangular array, as depicted in Figure 1. This construction is known as the *Shor code* [3], and it has the following properties:

- The *Z* information can be extracted from any 3 qubits in the same row.
  - Since the outer code is a *Z*-basis repetition code, each of its “qubits” (each row) alone carries the original *Z* information. But each of those “qubits” is encoded in the inner code, an *X*-basis repetition code, so all 3 qubits in the row are needed to extract the *Z* information.
- The *X* information can be extracted from any 3 qubits in 3 different rows.
  - Since the outer code is a *Z*-basis repetition code, all of its “qubits” (rows) are needed to extract the original *X* information. But since the inner code is an *X*-basis repetition code, any qubit in a given row carries the *X* information for that row.

Notice how these conditions are still “complementary” enough to prevent extracting both the *Z* and the *X* information from two disjoint sets of qubits. However, now we can lose up to 4 qubits without losing access to *either* the *Z* or the *X* information (as shown in Figure 1b), or we could use a majority vote to defend against an error on at least one unknown qubit when extracting either type of information.

But are there not other types of information in a qubit? Another surprising result is that if an encoding procedure (which can include losing or corrupting qubits afterward) preserves both the *Z* and the *X* information, then it in fact preserves *all* the information in the qubit. One way to understand this result is through a lens of quantum physics: if the *Z* information of the original qubit is represented by an observable in our available system, then we should be able to do not only *Z* measurements but also *Z rotations* by arbitrary angles (Although it may not be as *easy* to do such rotations as the corresponding measurements, especially if we want fault tolerance too!), and the same goes with *X* measurements and rotations. With arbitrary *Z* and *X* rotations, we have access to the entire Hilbert space of a qubit—a *logical qubit* encoded into multiple physical qubits. In fact, if we allow *controlled Z* and *X* rotations too, then we can move the state of the logical qubit to a single physical qubit again. Concretely, this means that if we encode a qubit into a Shor code and then corrupt an arbitrary qubit, there exists a physical procedure to perfectly recover the state of the original qubit—this is what it means for the Shor code to be a quantum error correction code.

Now that we are (hopefully) convinced that quantum error correction codes exist, we shall briefly mention an elephant in the room: Can all these procedures be done *fault-tolerantly*? The Shor code by itself only protects against a “static” error that occurs after encoding and before decoding, and if we want to operate on the logical qubit in any way, we must design our circuits very carefully in order not to become vulnerable to “dynamic” errors that occur during the procedure. Encoding (an arbitrary qubit) and decoding (Not to be confused with *syndrome decoding* (to be introduced later), which is a purely classical algorithm.) (to a physical qubit) are particularly problematic, due to the initial state of the former and the final state of the latter being vulnerable, but fortunately, for the purpose of quantum computation, we only really need initialization to specific states (such as |0〉 ) and logical measurements (outputting classical bits). Implementing arbitrary unitary gates fault-tolerantly is also difficult, but at least there is no fundamental obstacle. The fact that fault-tolerant quantum computation is indeed mathematically possible was first proved by Aharonov and Ben-Or in 1997 [4], but the scheme used in their proof is quite inefficient by modern standards. For now, we are actually going to forget the problem of fault tolerance and pretend that we can do quantum procedures perfectly when we want to. After we get familiar with the quantum error correction code we are going to use—not the Shor code, but the *surface code*—we will begin to tackle that problem in Section 5.

One of the advantages the surface code has over the Shor code is the ease of suppressing errors by *scaling up*, i.e., using more qubits. In our classical analogy, the repetition code is easy to scale up: as long as the error rate of each copy is less than 50% , by increasing the number of copies to do a majority vote with, the final error rate will be exponentially suppressed. We say that 50% is the *threshold* of the classical repetition code. For the Shor code, we can also try to increase the number of rows and columns in the qubit array, but there is a dilemma: increasing the number of columns (the size of the inner repetition code) is how we could suppress the probability that the *X* information is corrupted, but it also exponentially suppresses the probability that the *Z* information *survives* on each single row, requiring an exponential number of rows to compensate. A better idea is to nest the 9-qubit Shor code within itself (The technical term for such a nested code construction is *code concatenation*; the Shor code itself is a concatenation of a *Z*-basis repetition code and an *X*-basis repetition code.), but the resulting code family still has a relatively low threshold, and the nested structure also makes implementation hard, in general.

In comparison, the surface code can also be implemented on an d×d array of physical qubits, but it has a *Z*–*X* symmetry, and it can be scaled up simply by increasing *d*. The resulting code family has a relatively high threshold, up to 18.9% for depolarizing noise (Again, we are not considering fault-tolerant procedures yet, so this is just the threshold for “static” errors, often known as the *code-capacity* threshold. As we will see later, the threshold for “dynamic” circuit-level errors is on the order of 1% .) [5]. The surface code is also more “local” than the Shor code (in a sense that we will clarify later), making it easier to implement, especially for superconducting quantum hardware, which usually has limited qubit connectivity. The surface code is also quite flexible—the qubit lattice can be deformed, giving rise to the technique of *lattice surgery*, a well-established scheme for performing fault-tolerant quantum computation with the surface code. The surface code does have some disadvantages compared to some other codes, but those codes also have their own problems, so it is more of a trade-off situation. For now, the surface code remains a strong contender for the first generation of fault-tolerant quantum computers, and it should be a good option to focus on when learning modern quantum error correction.

We will start by briefly looking at some simple codes, such as the quantum repetition code and the Shor code, to get familiar with quantum codes, especially *stabilizer codes*, a class of codes with some nice properties. Then we will describe the surface code, and afterward we will show how one could build a fault-tolerant quantum computation scheme upon it, step by step. We will try to keep things simple: some methods that we describe may not be the most efficient known approach, but they will be adequate for demonstrating the theory. Finally, we will briefly discuss some open research problems.

## 2. Quantum Repetition Code

Recall that the requirements for the *Z*-basis quantum repetition code are:

- The *Z* information can be extracted from *any* one physical qubit.
- The *X* information can be extracted from *all n* physical qubits.

When the encoding procedure consists of adding ancilla qubits and then a unitary operation, the second requirement is satisfied for free since unitary operations are always reversible. To satisfy the first requirement, we want our procedure to map |0〉 to |00⋯0〉 , and |1〉 to |11⋯1〉 . This is readily implemented with n−1 CNOT gates, each between the original qubit and a new ancilla qubit initialized to |0〉 . For example, in the three-qubit case: (1)
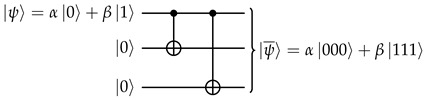


It is instructive to see what happens if we do a *Z* or *X* measurement on a single physical qubit:

- When measuring any physical qubit in the *Z* basis, the probabilities of getting the results |0〉 and |1〉 are ${\left|\alpha \right|}^{2}$ and ${\left|\beta \right|}^{2}$ , respectively, and afterwards the entire physical state collapses to $|\bar{0}\rangle =|000\rangle$ or $|\bar{1}\rangle =|111\rangle$ —this is essentially just a *Z* measurement on the logical qubit.
- To determine the effect of an *X* measurement, note that
(2) $$\begin{array}{cc} |\bar{\psi }\rangle & =\alpha |000\rangle +\beta |111\rangle \\ \; & =\left(\frac{\alpha }{\sqrt{2}}|+00\rangle +\frac{\alpha }{\sqrt{2}}|-00\rangle \right)+\left(\frac{\beta }{\sqrt{2}}|+11\rangle -\frac{\beta }{\sqrt{2}}|-11\rangle \right)\\ \; & =\left(\frac{\alpha }{\sqrt{2}}|+00\rangle +\frac{\beta }{\sqrt{2}}|+11\rangle \right)+\left(\frac{\alpha }{\sqrt{2}}|-00\rangle -\frac{\beta }{\sqrt{2}}|-11\rangle \right). \end{array}$$
  Therefore:
  - The probability of getting the result |+〉 is ${\left|\frac{\alpha }{\sqrt{2}}\right|}^{2}+{\left|\frac{\beta }{\sqrt{2}}\right|}^{2}=\frac{1}{2}$ , the same as the probability of getting |−〉 and independent of α and β (i.e., independent of the encoded state). Therefore the measurement did not directly yield any information about the logical qubit.
  - After the measurement, the remaining two qubits collapse into the state α|00〉±β|11〉 , with the sign of the second term depending on the measurement result. Therefore the logical qubit is still encoded, now in a two-qubit repetition code, but with a logical *Z* gate applied if the measurement result was |−〉 .

These results easily extend to quantum repetition codes of different lengths *n*, except when n=1 , where obviously both measurements directly measure the logical qubit.

A slightly surprising corollary is that we can extract the *X* information simply by measuring all physical qubits in the *X* basis. Each *X* measurement other than the final one shortens the repetition code, and if the result is |−〉 , applies a *Z* gate, which would always flip the *X* measurement result. The final *X* measurement measures the logical qubit, subject to these flips. Therefore, after compensating for these flips, the result of the logical *X* measurement should be determined by the *parity* of the physical *X* measurement results: if the number of |−〉 results is even (resp. odd), then the logical measurement result is |+〉 (resp. |−〉 ).

## 3. The Stabilizer Formalism

The encoding circuit (1) for the *Z*-basis repetition code is quite simple—it only has |0〉 ancilla qubits and CNOT gates. To obtain the encoding circuit for the *X*-basis repetition code, we can simply add Hadamard gates to the input and the output, or we can “absorb” them into the CNOT gates: (3)
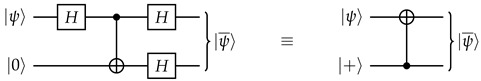
This way the ancilla qubit is initialized to |+〉 instead of |0〉 , but the circuit still has CNOT gates. Composing the encoding circuits for the two types of repetition circuits gives the encoding circuit for the Shor code, still only using |0〉 and |+〉 ancillas and CNOT gates. Since this simple class of circuits is capable of generating interesting quantum codes, it is worthwhile to study them thoroughly.

These circuits are in fact a subclass of a larger class of quantum circuits known as *stabilizer circuits*. A circuit is a stabilizer circuit if:

- All its unitary gates are *Clifford gates*.
  - Clifford gates are a group of unitary gates generated by the CNOT gate, the Hadamard gate, and the *S* gate (the π/2 phase gate).
- All its measurements are Pauli measurements.
- All newly introduced ancilla qubits are in *stabilizer states*. (We will define stabilizer states soon; for now, it suffices to know that |0〉 and |+〉 are stabilizer states.) (The definition of stabilizer circuits in [6] only includes Clifford gates and (computational basis) measurements, and any newly introduced ancilla qubits are covered by the condition that the input state must be ${|0\rangle }^{⊗n}$ . However, in the context of quantum error correction, it is often more natural to consider newly introduced ancilla qubits part of the circuit, and there is a symmetry between ancilla initialization and ancilla measurement.)

The well-celebrated Gottesman–Knill theorem [7] states that any stabilizer circuit can be classically simulated in polynomial time (assuming that every input qubit is initialized to |0〉 ). But more importantly, the proof of the theorem gives a powerful method to completely characterize the behaviors of stabilizer circuits, using the concept of *stabilizers*.

A *stabilizer* of an *n*-qubit state |ψ〉 is an *n*-qubit Pauli operator *P* such that P|ψ〉=|ψ〉 , i.e., |ψ〉 is an eigenvector of *P* with eigenvalue +1 . (If |ψ〉 is an eigenvector of *P* with eigenvalue −1 , then −P is a stabilizer.) Operationally, one can think of a stabilizer as a Pauli measurement with a consistent result on a certain state. For example, +Z stabilizes |0〉 , −Z stabilizes |1〉 , +X stabilizes |+〉 , and −X stabilizes |−〉 . Recall that multi-qubit Pauli operators can be measured by measuring each of their single-qubit factors and then multiplying the results ( ±1 ) together. For example, the logical *X* measurement procedure described in the last section is essentially measuring the multi-qubit Pauli $X^{⊗n}=X_{1}X_{2}\cdots X_{n}$ , and this implies that $\pm X_{1}X_{2}\cdots X_{n}$ stabilizes the logical *X* eigenstates, $|\bar{\pm }\rangle =|00\cdots 0\rangle \pm |1\cdots 1\rangle$ .

It is easy to verify that the set of all stabilizers of a state forms an Abelian subgroup of the Pauli group $P_{n}$ , called the *stabilizer group*. All stabilizers must be Hermitian (i.e., with prefactor ±1 instead of ±i ), and thus each non-identity element of the stabilizer group has order 2, so the size of the stabilizer group is always of the form $2^{k}$ , where *k* is the number of independent generators of the group. The maximum value of *k* is *n*; when k=n , the stabilizer group completely determines the state up to a phase factor, and such a state is known as a *stabilizer state*.

The Gottesman–Knill theorem makes use of a fundamental property of Clifford gates: A Clifford gate *C* satisfies $\forall P\in P_{n},\;CPC^{†}\in P_{n}$ . Since *C* is unitary, $P\mapsto CPC^{†}$ is a permutation over $P_{n}$ . For any given state |ψ〉 , $CPC^{†}$ is a stabilizer of C|ψ〉 if and only if *P* is a stabilizer of |ψ〉 . Therefore, Clifford gates preserve the size of the stabilizer group, and, in particular, stabilizer states remain stabilizer states. Furthermore, these stabilizers can be tracked efficiently, giving an effective method to characterize the behavior of a Clifford circuit.

**Example.** We will demonstrate this technique on the encoding circuit (1) for the 3-qubit *Z*-basis quantum repetition code. Initially, both ancilla qubits are in the state |0〉 , so $+Z_{2}$ and $+Z_{3}$ are both stabilizers of the initial state. Although |ψ〉 is an arbitrary state, in general, for the purpose of characterizing the behavior of the circuit, it suffices to consider the cases where it is an arbitrary stabilizer state. Therefore, we assume that either $\pm Z_{1}$ or $\pm X_{1}$ is an initial stabilizer and track both cases simultaneously (A possibly mathematically “cleaner” trick is to let |ψ〉 be half of a Bell pair. Then the initial stabilizers will include $Z_{0}Z_{1}$ and $X_{0}X_{1}$ , and since none of our operations will touch $Z_{0}$ and $X_{0}$ , the results we get will be equivalent to what we get in the main text.). The initial state is thus described by:

(4) $$P_{1}=+Z_{2},\;P_{2}=+Z_{3},\;\bar{Z}=+Z_{1},\;\bar{X}=+X_{1}.$$

Now we process the first gate in the circuit, a CNOT gate with qubit 1 as the control and qubit 2 as the target. Such a CNOT gate maps the stabilizers as follows:

(5) $$Z_{1}\mapsto Z_{1},\;X_{1}\mapsto X_{1}X_{2},\;Z_{2}\mapsto Z_{1}Z_{2},\;X_{2}\mapsto X_{2}.$$

Note that although the entire *n*-qubit Pauli group has $4\;\cdot \;4^{n}$ elements, we only need these 4 entries to fully describe the behavior of a two-qubit Clifford gate. The main reason is that, since $CPQC^{†}=\left(CPC^{†}\right)\left(CQC^{†}\right)$ , the map $P\mapsto CPC^{†}$ preserves the group structure of the Pauli group, so we only need to specify the action of the map for a set of generators. The scalar factors ( ±1 , ±i ) are always mapped to themselves, as are the Pauli operators on untouched qubits ( $Z_{3}$ , $X_{3}$ ), so we can further reduce the specification to $Z_{1}$ , $X_{1}$ , $Z_{2}$ , and $X_{2}$ .

Applying the mapping (5) to (4) gives:

(6) $$P_{1}=+Z_{1}Z_{2},\;P_{2}=+Z_{3},\;\bar{Z}=+Z_{1},\;\bar{X}=+X_{1}X_{2}.$$

The CNOT gate from qubit 1 to qubit 3 can then be processed in the same way, giving the final result:

(7) $$P_{1}=+Z_{1}Z_{2},\;P_{2}=+Z_{1}Z_{3},\;\bar{Z}=+Z_{1},\;\bar{X}=+X_{1}X_{2}X_{3}.$$

The reader may notice that the last equation confirms the final result of the last section: if the initial state of |ψ〉 is |±〉 , the initial stabilizers will include $\pm X_{1}$ , and the final stabilizers will include $\pm \bar{X}=\pm X_{1}X_{2}X_{3}$ . Therefore we can determine the sign of this stabilizer (i.e., do a logical *X* measurement) by measuring all physical qubits in the *X* basis and taking the parity of the results.

**Figure 2:**
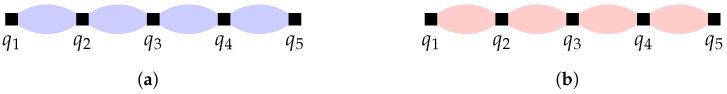
“Canonical” stabilizer generators of the 5-qubit (**a**) *Z*-basis and (**b**) *X*-basis repetition codes. The labels $q_{1}$ – $q_{5}$ indicate physical qubits. Each individual tinted area represents a stabilizer, with blue ones indicating products of *Z* Pauli operators and red ones indicating products of *X* Pauli operators. Thus the sets of stabilizers depicted are $\left\{Z_{1}Z_{2},Z_{2}Z_{3},Z_{3}Z_{4},Z_{4}Z_{5}\right\}$ and $\left\{X_{1}X_{2},X_{2}X_{3},X_{3}X_{4},X_{4}X_{5}\right\}$ , respectively.

We should now remind the reader that what we obtain is a set of *generators* of the stabilizer group (more precisely, there are four sets of generators for four different scenarios— $\left\{P_{1},P_{2},\pm \bar{Z}\right\}$ when the initial stabilizers include $\pm Z_{1}$ and $\left\{P_{1},P_{2},\pm \bar{X}\right\}$ when the initial stabilizers include $\pm X_{1}$ ), and thus there are multiple equivalent representations. For example, $P_{1}P_{2}=+Z_{2}Z_{3}$ is also an unconditional stabilizer that could replace either $P_{1}$ or $P_{2}$ . In fact, $\left\{Z_{1}Z_{2},Z_{2}Z_{3},\ldots ,Z_{n-1}Z_{n}\right\}$ is often used as a “canonical” set of stabilizer generators for the *n*-qubit repetition code. It has a nicer geometric representation (see Figure 2) when the qubits are arranged in a chain, which can translate to easier implementation on actual quantum hardware.

Similarly, $P_{1}\bar{Z}=+Z_{2}$ , $P_{2}\bar{Z}=+Z_{3}$ , and even $P_{1}P_{2}\bar{Z}=+Z_{1}Z_{2}Z_{3}$ are all stabilizers whenever $\bar{Z}$ is, and denoting any of them as $\bar{Z}$ is equally valid. Even $\bar{X}$ can be replaced with $P_{1}\bar{X}=-Y_{1}Y_{2}X_{3}$ (Note that the minus sign is due to the factor *i* in the identity ZX=iY .), etc., while we will often simply refer to $\left\{P_{k}\right\}$ and $\left\{\bar{Z},\bar{X}\right\}$ as *code stabilizers* and *logical Z/X operators*, respectively, sometimes we will instead refer to them as code stabilizer *generators* and logical operator *representatives*, to emphasize this freedom.

**Stabilizer codes and CSS codes.** All quantum codes whose encoding circuits involve only |0〉 and/or |+〉 ancilla qubits and Clifford gates can be described in this way, and such codes are known as *stabilizer codes*. If all the Clifford gates are CNOT gates, then the code will have some extra special properties: each stabilizer generator and each logical representative will consist of either only *X* factors or only *Z* factors. Such codes are known as *Calderbank–Shor–Steane (CSS) codes*. The quantum repetition code, the Shor code, and the surface code (which we will introduce later) are all CSS codes.

To facilitate analyzing the error correction capabilities of stabilizer codes, we will make an important simplification: we will assume that all errors that are going to occur are stochastic Pauli errors, i.e., unintended Pauli *X*, *Y*, or *Z* gates are applied to qubits with low probabilities according to some classical probability distribution (possibly with some correlation). Although such error models might seem very limited, *qualitatively* (Essentially, this means that given a general error model, one can in theory give a stochastic Pauli error model that is as “difficult” as the original error model. However, the physical error rate of the latter model may be somewhat larger than the original model, and in practice such a *quantitative* difference may still be a big problem!) they are actually adequate for capturing all possible forms of quantum errors. One may find this statement more plausible by noting that a maximal depolarizing channel that replaces any qubit state with the maximally mixed state can be represented as a uniform mixture between {I,X,Y,Z} ; therefore, in a sense, Pauli errors can span the space of all possible errors. For a rigorous proof that covers the case of coherent errors (such as an error process that always occurs but only rotates the qubit by a tiny angle), we refer the reader to [8].

The effect of a given Pauli error on a stabilizer code can be very easily analyzed: Pauli gates are special cases of Clifford gates that satisfy $QPQ^{†}=\pm P$ , where the sign is plus if *P* and *Q* commute and minus if they anti-commute. Therefore, a Pauli error does not affect the Pauli string part of stabilizers but only flips their signs if they anticommute with the error. As a special case, if the Pauli error is the same as a code stabilizer, then it commutes with all code stabilizers and all logical operators, meaning that it does not affect the state at all, and such an “error” can be freely ignored. This is to be expected, since P|ψ〉=|ψ〉 is the definition of a stabilizer in the first place!

Another special case is when the Pauli error is the same as a logical operator representative, such as $\bar{Z}$ . Such an error also does not affect the code stabilizers, but it will flip the sign of $\bar{X}$ , meaning that $|\bar{+}\rangle$ becomes $|\bar{-}\rangle$ and vice versa. In fact, this is exactly how we would apply a *Z* gate to the logical qubit. But this happening as an error is a bad thing, since the error will corrupt the logical information with no way for us to even notice. Unfortunately, this can easily happen with the repetition code, because the logical operator $\bar{Z}=Z_{1}$ has length 1 (i.e., it is only supported on a single qubit), and in fact every single-qubit *Z* is a logical *Z* representative. This confirms our observation that the *Z*-basis repetition code does not preserve the *X* information well. In general, we want all logical operator representatives (Note that this also includes the logical *Y* operator $\bar{Y}=i\bar{X}\bar{Z}$ , although for CSS codes it is never shorter than either $\bar{Z}$ or $\bar{X}$ . For codes encoding multiple logical qubits, logical operators that span multiple logical qubits should also be considered.) of our quantum codes to be long, so that, barring very long-range correlations, the probability that such an undetectable error happens will be exponentially small. The length of the shortest logical operator representative of a quantum code is known as the *distance d* of the code.

**Figure 3:**
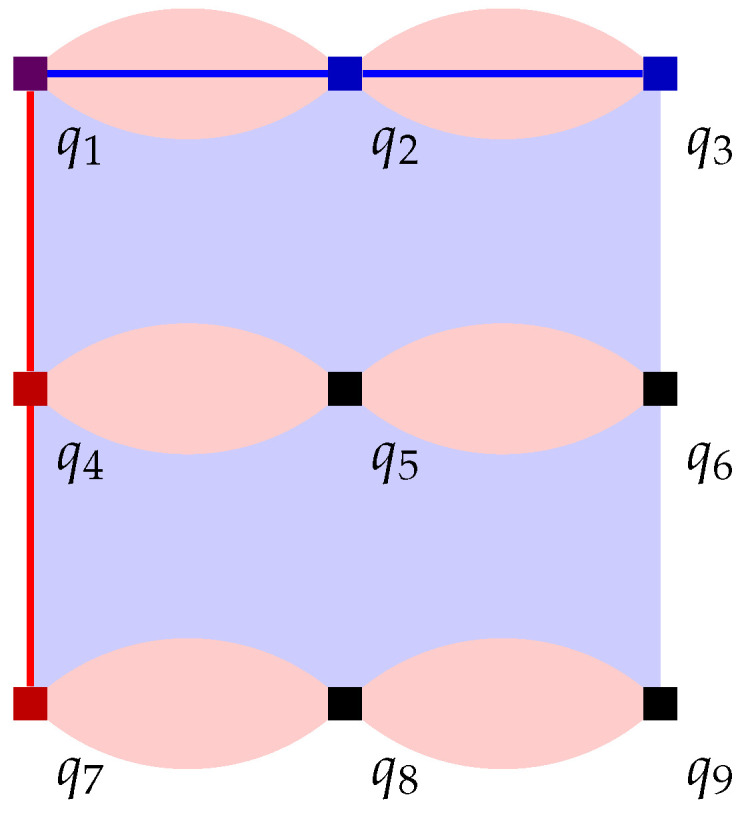
Stabilizer generators and logical operators of the 9-qubit Shor code. Tinted areas represent stabilizers, and the blue and red solid lines represent the *Z* and *X* logical operators. Two stabilizers and/or logical operators commute with each other if they are both blue or both red (*Z* commutes with *Z* and *X* commutes with *X*), or if they overlap at an even number of qubits. The reader can check that in this diagram, every stabilizer commutes with every other stabilizer and every logical operator, but the two logical operators anti-commute because they overlap at exactly one qubit.

The Shor code is an example of a quantum code with distance 3. As illustrated in Figure 3, one can write down its stabilizers and logical operators simply by following the concatenated code structure. First, the stabilizers of the inner *X*-basis repetition code:

(8) $$X_{1}X_{2},\;X_{2}X_{3},\;X_{4}X_{5},\;X_{5}X_{6},\;X_{7}X_{8},\;X_{8}X_{9}.$$

Then, the stabilizers of the outer *Z*-basis repetition code, encoded in inner code logical operators:

(9) $$\bar{Z_{1}}\bar{Z_{2}}=Z_{1}Z_{2}Z_{3}Z_{4}Z_{5}Z_{6},\;\bar{Z_{2}}\bar{Z_{3}}=Z_{4}Z_{5}Z_{6}Z_{7}Z_{8}Z_{9}.$$

Finally, the overall logical operators:

(10) $$\bar{Z}=\bar{Z_{1}}=Z_{1}Z_{2}Z_{3},\;\bar{X}=\bar{X_{1}}\bar{X_{2}}\bar{X_{3}}=X_{1}X_{4}X_{7}.$$

To verify that the code indeed has distance 3, we need to check that *every* logical operator representative has at least length 3. One advantage of CSS codes is that we only need to try combining $\bar{Z}$ with *Z* stabilizers and $\bar{X}$ with *X* stabilizers, since combining a *Z*-only Pauli string with an *X*-only Pauli string can never reduce the length. With a little effort, one can show that *Z*-only logical operators are always an odd number of whole rows, and *X*-only logical operators always contain an odd number of qubits in each row, thus proving the code distance. The code distance can be increased by increasing the number of rows and columns (i.e., the size of the outer code and the inner code): with an n×m qubit array, the code distance will be d=max(n,m) .

**Stabilizer measurements.** Pauli errors that are neither code stabilizers nor logical operators must anti-commute with at least one stabilizer, so they should at least be detectable. In fact, the stabilizer formalism gives a conceptually easy way to detect errors: just measure the values of the stabilizers.

Previously we have mentioned that multi-qubit Pauli operators can be measured by measuring each of their single-qubit factors, but this procedure is destructive and thus only appropriate if we are measuring the logical qubit anyway. (It happens implicitly when, for example, we measure every physical qubit of a *Z*-basis repetition code in the *Z* basis and determine the measurement result with a majority vote). Instead, we can use additional ancilla qubits to measure a stabilizer non-destructively, keeping the code state intact.

The circuit below demonstrates the technique, performing a non-destructive ZZ measurement on qubits $q_{1}$ and $q_{2}$ . This method is straightforward to generalize to more qubits. To measure stabilizers with *X* and/or *Y* components, we can sandwich the CNOT gates with Hadamard and/or HS gates as appropriate.(11)
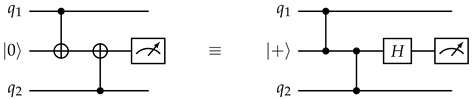


Of course, any quantum measurement that yields information must disrupt the state to some extent. From the perspective of qubits $q_{1}$ and $q_{2}$ , this circuit has a 50% probability to apply a Pauli ZZ gate (which may be more evident from the alternate form of the circuit on the right side). However, unlike measuring each qubit individually, it never applies only a single *Z* gate on either $q_{1}$ or $q_{2}$ , so the values of Pauli operators such as XX , which commute with ZZ but not with ZI or IZ , are preserved.

The good news is that measuring a stabilizer this way never disturbs a valid code state: As mentioned above, applying a stabilizer Pauli as a gate by definition does not change the state. If this stabilizer has been flipped by a Pauli error, then applying this Pauli gate would just multiply the state by the global phase −1 , which still has no observable effect. The only situation where this disturbance matters is when the state before measurement is not an eigenstate of the stabilizer; however, after measurement, the stabilizer value will collapse to ±1 anyway. In a sense, the stabilizer measurement is forcing an arbitrary error to “behave like a Pauli error”, which is part of the reason why an error model with only Pauli errors is adequate.

Ignoring errors during the measurement procedure, measuring any set of stabilizer generators is enough to detect all detectable errors. In practical implementations, we usually choose stabilizer generators that are low-weight and work well with the connectivity constraints of the hardware. For example, if we want to implement a repetition code on a chain of qubits, we may want to take the stabilizers $\left\{Z_{1}Z_{2},Z_{2}Z_{3},\ldots ,Z_{n-1}Z_{n}\right\}$ (Another advantage of this set of stabilizer generators is that each qubit is only involved a constant number of times, allowing a low-depth circuit to measure all the stabilizers.).

We can also try to correct any errors that we have detected, although we will often be faced with ambiguities since many Pauli errors can cause the same stabilizer flip pattern (often known as the *error syndrome*). For example, for the 3-qubit *Z*-basis repetition code, $E_{1}=X_{1}$ and $E_{2}=X_{2}X_{3}$ cause exactly the same stabilizer flips. If we decide to correct this syndrome as $E_{1}$ , but what has actually occurred is $E_{2}$ , then the overall effect of the error and the correction will be $E_{1}E_{2}$ . This will be a problem only when $E_{1}E_{2}$ is a logical operator, but in this case the length of $E_{1}E_{2}$ must be at least *d*, and at least one of $E_{1}E_{2}$ will have length >⌊(d−1)/2⌋ . Therefore, for any distance-*d* code, there is a way to choose corrections to guarantee correcting up to ⌊(d−1)/2⌋ errors.

## 4. Surface Code

One of the problems with scaling up the Shor code is that the size of stabilizer generators does not remain constant. In contrast, the surface code is a stabilizer code generated by stabilizers with length at most 4, and these stabilizer generators are spatially local, making it much easier to implement in practice. The surface code also handles *Z* and *X* errors symmetrically, allowing it to achieve a better overall error correction performance.

**Figure 4:**
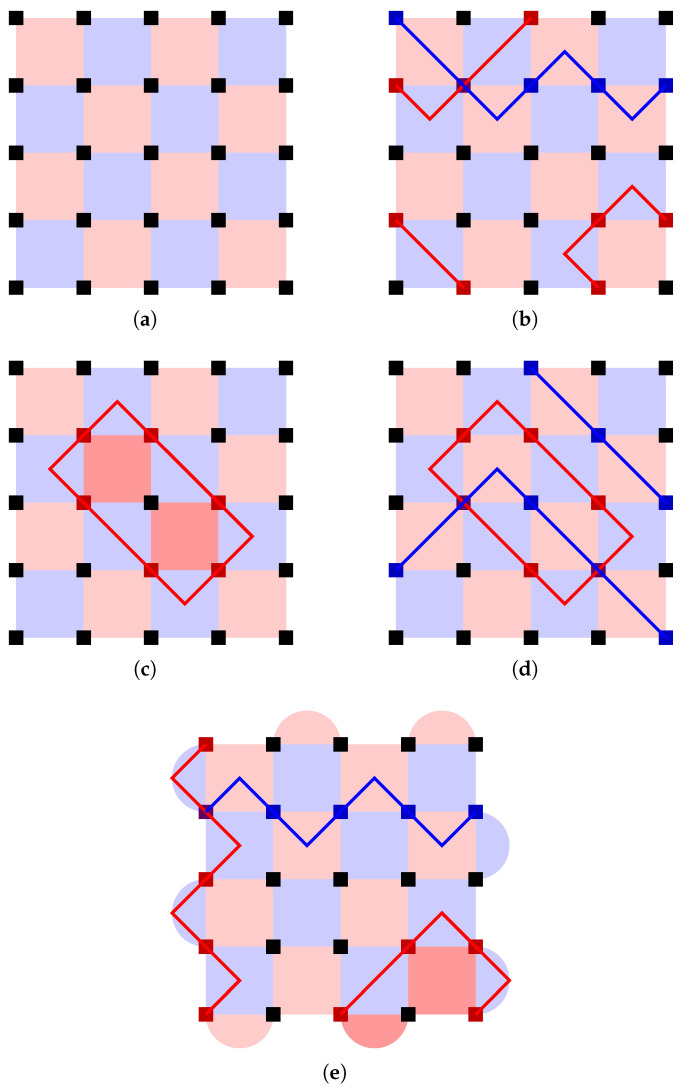
The surface code. (**a**) Tiling the plane with *Z* and *X* stabilizers. As in Figure 3, vertices represent physical qubits, blue-tinted squares represent *Z* stabilizers, and red-tinted squares represent *X* stabilizers. (**b**) Some ways surface code logical operators could zigzag through the grid while satisfying the commutation relation with (the opposite type of) stabilizers. Blue strings represent *Z* Pauli operators, and red strings represent *X* Pauli operators. (**c**) Closed loops form stabilizers instead of logical operators. The *X*-type Pauli operator represented by this loop is a stabilizer, since it is exactly equal to the product of the two highlighted *X* stabilizer generators. (**d**) Any valid blue string must intersect the red closed loop an even number of times and, thus, commute with the closed string. (**e**) Adding boundary stabilizers gives a complete d=5 surface code.

The basic idea of surface codes is to tile a plane with two colors of squares in a checkerboard pattern, as depicted in Figure 4a. Here, the vertices represent physical qubits, the blue squares represent *Z* stabilizers, and the red squares represent *X* stabilizers. We first verify that such a construction indeed yields a set of commuting stabilizers:

- Two orthogonally adjacent squares have different colors and thus different stabilizer types (*Z* or *X*), but they share two vertices, so the stabilizers they represent commute with each other.
- Two diagonally adjacent squares have the same type, so although they only share one vertex, they also commute without problem.
- Any other two squares share no vertices, so their stabilizers trivially commute.

The stabilizers depicted in Figure 4a do not yet form a good code, but it is instructive to analyze the properties of the logical operators allowed by this stabilizer configuration. As with other CSS codes, we can consider the *Z*-type and *X*-type logical operators separately. One important property of the surface code is that:

*Except on boundaries, any single-qubit Z or X Pauli always flips exactly two stabilizer generators.*

To make use of this property, we draw each single-qubit *Z* or *X* Pauli as a diagonal line segment that connects the center points of the two stabilizers flipped. When we draw a logical operator this way, each stabilizer must be flipped an even number of times, so the number of such line segments meeting at the center of each square must be 0, 2, or 4. Therefore, the logical operator must consist of one or more “strings” that are never interrupted in the middle of the lattice and only begin and end on the boundaries. (When 4 line segments meet at the center of a square, they can be separated into two strings.) Figure 4b shows some examples of such strings. Note that red strings are always drawn on top of blue stabilizers and vice versa because *X* errors flip *Z* stabilizers and vice versa. When a red string intersects a blue string, it must be a perpendicular intersection on a qubit, as illustrated by the two upper strings in Figure 4b.

Instead of starting and ending at boundaries, a string can also form a closed loop in the middle of the lattice, as depicted in Figure 4c. The Pauli operator represented by such a closed loop actually commutes with all valid Pauli strings: All red strings commute with each other since *X* commutes with *X*, and any blue string must intersect a closed red loop an even number of times—as demonstrated in Figure 4d—and thus commute with it anyway. Therefore, this “closed-loop” Pauli operator cannot be an *X* logical operator of a logical qubit, since there is no *Z* logical operator that could anti-commute with it. Instead, it must be a code stabilizer. Indeed, close inspection of the closed string in Figure 4c shows that it is in fact the product of the two highlighted *X* stabilizer generators.

The fact that not all valid strings are (non-trivial) logical operators is a good thing, because, as mentioned before, a logical operator is also an undetectable logical error, and thus we want to constrain them: A good code (one with large code distance) is one where all logical operators are long. The reason Figure 4a is not yet a good code is that the logical operators are free to begin and end at any boundaries. Taking a hint from the logical operators of the Shor code, we want our *Z* logical operators to be “row-like” and *X* logical operators to be “column-like”: the former should only go from the left boundary to the right boundary, and the latter should only go from the top boundary to the bottom boundary.

Such boundary constraints can be enforced by adding some *X* stabilizers on the top and bottom boundaries and some *Z* stabilizers on the left and right boundaries, as depicted in Figure 4e. Now each *X* Pauli flips exactly two stabilizers except on the *top and bottom* boundaries, so an *X* string cannot simply “escape” from the left or right boundary. It can begin and end at the same boundary, like illustrated in the lower right corner of Figure 4e; however, since a *Z* logical operator would not be able to escape between its endpoints, such a string will turn out to be a stabilizer rather than a logical operator. Therefore, all *X* logical operators indeed need to go from the top boundary to the bottom boundary at least once, and their lengths are lower bounded by the number of rows in the grid. Similarly, *Z* logical operators go from the left to the right, with lengths lower bounded by the number of columns. With a d×d qubit array, we have thus shown that we have a distance-*d* surface code.

The surface code we have described encodes exactly one logical qubit: the product of any two *Z* logical operators will be a stabilizer, as is the product of any two *X* logical operators. The topology of the square lattice ensures that a given *Z* logical operator and a given *X* logical operator must intersect, and in fact they must intersect at an odd number of qubits. The fact that there is exactly one logical qubit can also be shown by counting stabilizer generators: There are ${\left(d-1\right)}^{2}$ square stabilizers and 2(d−1) boundary stabilizers (This is easier to see for the case shown in Figure 4e where *d* is odd (which is much more commonly studied anyway since the minimum distance to guarantee correcting *t* errors is 2t+1 ), but also true when *d* is even, even though in that case one of the stabilizer types (*Z* or *X*) will have 2 more boundary stabilizers than the other type.) on $d^{2}$ physical qubits, leaving space for exactly $d^{2}-{\left(d-1\right)}^{2}-2\left(d-1\right)=1$ logical qubit.

**Decoding and threshold.** Our surface code encodes 1 logical qubit into $d^{2}$ physical qubits, with distance *d*. These are the same parameters as the (generalized) Shor code, and these parameters alone do not guarantee that the code family has a threshold. The reason is evident when we consider what happens when we scale up the code under a constant error rate *p*: The expected number of errors $pd^{2}$ increases faster than *d*, so the fact that the code can always correct up to ⌊(d−1)/2⌋ errors does not mean much.

However, the code distance does not fully describe the error correction capability of a quantum code. For example, the d×d generalized Shor code can easily correct up to ${\left\lfloor \left(d-1\right)/2\right\rfloor }^{2}$ errors if they all occur in the same ⌊(d−1)/2⌋ rows, with up to ⌊(d−1)/2⌋ errors per row. In general, an error configuration $E_{1}$ is only damaging if there exists another error configuration $E_{2}$ that “seems more likely” and such that $E_{1}E_{2}$ is a logical operator. Compared to the Shor code, the surface code allows more freedom with its *Z* logical operators but is more restrictive with its *X* logical operators, achieving a better balance, so we could hope that a more careful analysis will show that the surface code does have a threshold.

Note that when we stated that a distance-*d* code can correct up to ⌊(d−1)/2⌋ errors, we implicitly assumed that we will always choose a correction with minimum weight among all possible corrections consistent with the observed error syndromes. The procedure to choose a correction based on error syndromes is known as *decoding*, and a decoder that satisfies this assumption is a *minimum-weight decoder*. There are actually a few inaccuracies with a strictly minimum-weight approach, but we will talk about them later, mainly in Section 6.2. For now, we just note that the error correction performance of a code, including its threshold, can depend on the choice of decoder.

In fact, minimum-weight decoding for the surface code is already a computationally hard problem, especially when we take fault tolerance into account later. Therefore, people often make another simplification by separating the error syndrome into *Z* syndromes and *X* syndromes and decoding them separately. The CSS structure of the surface code means that the set of valid *X* corrections is independent of *X* syndromes (recall that *X* errors flip *Z* syndromes), and the same is true with *Z*. However, by decomposing the problem this way, we change the goal function: a single *Y* correction is decomposed into a *Z* correction and an *X* correction, adding 2 to the total correction weight instead of 1.

The advantage of such a decomposition is that decoding a single type of syndrome can be reduced to the problem of *minimum-weight perfect matching* (MWPM), which has an efficient (classical) algorithm. Since each single-qubit *Z* or *X* Pauli flip affects at most two stabilizer generators, we can build a *decoding graph* with qubits as edges and stabilizers as vertices. Some errors on the boundary only flip a single stabilizer generator, which can be handled by connecting the other end of its edge to a special “boundary vertex”, which is flipped if and only if an odd number of other vertices are flipped (This can be interpreted as adding the *product of all the stabilizer generators* to the set of stabilizer generators. Each single-qubit *Z* or *X*, on the boundary or not, flips exactly two stabilizers in this new set.). A valid error configuration is then represented as a set of edges that constitute a *perfect matching* of flipped vertices, and the goal becomes to find a minimum-weight perfect matching, which can be solved by the well-known blossom algorithm (Strictly speaking, the problem we have just described is a variant of the original MWPM problem. It has a polynomial-time reduction to the original MWPM problem but can be solved faster with the *sparse blossom* algorithm introduced in [9], which is the algorithm PyMatching 2 actually implements.). PyMatching 2 [9] is an optimized implementation of the MWPM decoder, and it is now almost an indispensable tool in every quantum error correction researcher’s toolkit.

It is shown in [10] that the surface code indeed has a threshold when decoded with an MWPM decoder. The key observations of the proof are (Adapted to the case of code-capacity threshold; the original proof focuses on the stronger circuit-level error model, which we will introduce later.):

- If MWPM decodes an error configuration E “incorrectly” (in the sense that the difference is a logical operator), then there must exist a *single-string* logical operator of length *l* that overlaps with E on at least l/2 qubits.
- The number of such strings with length *l* can be upper bounded by $d\;\cdot \;3^{l}$ , since there are *d* possible starting points and then we take *l* steps, each with at most 3 choices.
- For a fixed single-string logical operator, the probability that E overlaps with it on at least l/2 qubits is upper bounded by $2^{l}\;\cdot \;p^{l/2}$ .
- When $6\sqrt{p}<1$ , i.e., when p<1/36 , the series
(12) $$\sum_{l=d}^{\infty }d\;\cdot \;3^{l}\;\cdot \;2^{l}\;\cdot \;p^{l/2}=\sum_{l=d}^{\infty }d\;\cdot \;{\left(6\sqrt{p}\right)}^{l}$$
  converges to $O\left(d\;\cdot \;{\left(6\sqrt{p}\right)}^{d}\right)$ .

We note, however, that the result 1/36 is only a loose theoretical lower bound for the threshold. In general, researchers estimate the thresholds for different codes different decoders under different error models through Monte Carlo simulation, which is thankfully easy for stochastic Pauli error models because of the following: (1) stabilizer circuits can be efficiently classically simulated and (2) one can precompute the effects of all possible single-qubit Pauli errors to make the rest of the simulation completely classical. Stim [11] is a well-optimized simulator that can perform both tasks, making it another widely recognized tool in quantum error correction research.

## 5. Fault Tolerance

In the above sections, we have been ignoring any errors that may occur when performing operations on physical qubits, especially during the stabilizer measurement procedure. In this section, we will first describe a method to overcome errors during stabilizer measurements through repetition and preserve a logical qubit for an extended period of time, a procedure that can be experimentally tested with the *memory experiment*. Then we will discuss *lattice surgery* [12], a technique that is in some sense a natural extension of the memory experiment but allows execution of arbitrary logical Clifford gates. Finally, we will show how *magic state injection* and *magic state distillation* enable performing non-Clifford gates with lattice surgery, thus achieving universal fault-tolerant quantum computation.

### 5.1. Memory Experiment

**Motivating example.** We will first use a different idealized situation to illustrate the general technique that we will use. Imagine that we have a system where all qubits and unitary gates are perfect, but each measurement has a probability to get a flipped result. To measure a qubit fault-tolerantly, we can then make use of repeated non-destructive measurements: (13)
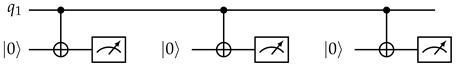


Obviously, we should do a majority vote to get the final measurement result. However, similar to how we could reframe the majority vote (between qubits) for the quantum repetition code in terms of stabilizers, we also want to reframe this “temporal” majority vote in some way in order to better integrate it into our surface code decoding framework later.

Denote the measurement results (0 or 1) as $r_{1},r_{2},\ldots ,r_{m}$ . Inspired by the stabilizers of the repetition code, we take the XOR of consecutive measurement results: $\Delta_{i}=r_{i}⊕r_{i+1}$ for i=1,2,…,m−1 . In the absence of errors, $\Delta_{i}$ should be 0 no matter what the measurement result is, and in this aspect $\Delta_{i}$ is indeed somewhat similar to stabilizers. In the decoding literature, they are known as *detectors*.

We now examine the effects of measurement errors on the detectors. For 2≤i≤m−1 , an error on $r_{i}$ flips exactly two detectors, $\Delta_{i-1}$ and $\Delta_{i}$ . An error on $r_{1}$ only flips $\Delta_{1}$ , and an error on $r_{m}$ only flips $\Delta_{m-1}$ . Since each error flips at most two detectors, this is a “graph-like” decoding problem that can be solved with MWPM. The decoding graph will be a cycle (with the boundary vertex connected to both $\Delta_{1}$ and $\Delta_{m-1}$ ), so there will be exactly two feasible matchings, and this is indeed just a more convoluted way to implement a majority vote.

**Quantum memory.** However, the idea of defining the XOR of consecutive measurement results as detectors will become more useful when applied to repeated stabilizer measurements. When trying to preserve a logical qubit for an extended period, we already have an incentive to repeat stabilizer measurements due to idle errors on the physical qubits. If we keep the physical qubit idling, idle errors will accumulate over time and eventually (The time needed is short by human standards (e.g., usually less than 100 μs for superconducting qubits), but thankfully still long enough for the quantum hardware to apply many quantum operations.) go over the code threshold. However, if we repeatedly measure all stabilizers when the accumulated idle error is still well below the threshold, then the probability of a decoding failure is exponentially small in *d*, and on average we can preserve the qubit for an exponentially long time. Therefore, this procedure can be worthwhile even if it introduces some new error on top of the idle error.

When there is a possibility of measurement errors, we can no longer decode each round of stabilizer measurements with the same decoding graph as before, because a measurement error only flips a single stabilizer measurement result, a kind of error not accounted for in the original decoding graph. Even if we try to add edges corresponding to measurement errors to the decoding graph, we will get undesirable behaviors, such as the decoder believing a stabilizer flip to be a measurement error even though it has persisted for many rounds, which would greatly impact the error correction performance. It is also not quite appropriate to do simple majority votes between the measurement results of many rounds, because it is possible that the stabilizer was actually flipped during these rounds. However, the concept of detector allows one to combine surface code decoding and measurement error correction in an elegant way.

As before, we define detectors $\Delta_{P,i}$ as $r_{P,i}⊕r_{P,i+1}$ , i.e., the XOR of consecutive measurement results on the same stabilizer *P*, and analyze how each type of error affects detectors. For simplicity, we will first limit our analysis to two error types—qubit idle errors between stabilizer measurement rounds and measurement errors during a stabilizer measurement round. This is known as the *phenomenological error model* in the literature.

- A measurement error in round *i* on the stabilizer *P*, as before, will flip the detectors $\Delta_{P,i-1}$ and $\Delta_{P,i}$ , unless round *i* is the first or the last round, in which case only one of the two exists.
  - When we implement the stabilizer measurement with a circuit like (11), such an error is usually (but not always) caused by an error on the ancilla qubit for this stabilizer, so this kind of error is also known as *ancilla errors*.
- A *Z* or *X* error between round *i* and round i+1 will flip the values of up to two stabilizers $P_{1}$ and $P_{2}$ , which can be observed starting from round i+1 . (A *Y* error can be decomposed into a *Z* error and an *X* error and handled as such.) In the absence of other errors, these stabilizers will remain flipped, so the only *detector* flips caused by this error are $\Delta_{P_{1},i}$ and $\Delta_{P_{2},i}$ .
  - To differentiate from ancilla qubits, the physical qubits that directly encode the logical qubit are usually referred to as *data qubits*, and thus this kind of error is also known as *data errors*.

In both cases, up to two *detectors* are flipped. Therefore, we can build a decoding graph for the entire procedure, which will become three-dimensional: for an experiment with *m* rounds, there will be m−1 layers of detectors, each layer with the same edges as the original decoding graph of the surface code (corresponding to *Z* or *X* errors between rounds), and between consecutive layers, vertices at the same location are connected (corresponding to measurement errors). When drawing the decoding graph, the time axis is often drawn vertically, so the edges of the first type (within a layer) are called *horizontal edges,* and edges of the second type (between layers) are called *vertical edges*.

We can again decode with MWPM on this decoding graph to get a “correction” (a set of possible errors) from the observed detector values, but unlike in our motivating example, we cannot expect this set to exactly match the set of errors that actually happened with high probability, because the decoding graph now contains many short cycles, leading to likely ambiguities for the MWPM decoder. This is already the case for surface code decoding, where these short cycles correspond to stabilizers and, thus, do not affect the logical state. In our 3D decoding graph, there are two types of “basic” short cycles:

- One type is short cycles within a layer, which still correspond to stabilizers.
- The other type of short cycle spans two consecutive layers and consists of (up to) 4 edges—two horizontal edges that correspond to the same edge in the original decoding graph and two vertical edges that connect the endpoints.
  - Physically, this means that an error flips two stabilizers, but after one stabilizer measurement round, the same error happens again, flipping the same two stabilizers; and in that stabilizer measurement round in-between, both these stabilizers happen to be measured incorrectly, leaving no visible trace.

Obviously, in the second case the error has corrected itself, so we do not need to do anything about it. This also means that we do not care if such a cycle is the difference between the actual error configuration and our correction. For example, suppose that $P_{1}$ and $P_{2}$ are two adjacent stabilizers, and the set of non-zero detectors we observe is $\left\{\Delta_{P_{1},1},\Delta_{P_{2},2}\right\}$ , meaning that the measurement results of both $P_{1}$ and $P_{2}$ have flipped once each, but $P_{2}$ flipped one round later than $P_{1}$ . This can be interpreted in two ways:

- The qubit error that flips $\left\{P_{1},P_{2}\right\}$ occurred after round 1, but $P_{2}$ was measured incorrectly in round 2, thus not properly reflecting the flip until round 3.
- The qubit error that flips $\left\{P_{1},P_{2}\right\}$ occurred after round 2, but $P_{1}$ was measured incorrectly in round 2, thus seemingly flipping one round earlier.

We cannot distinguish between these two cases, but fortunately we do not need to.

However, there is a problem with the *time boundary* of the decoding graph, i.e., the first round and the last round of stabilizer measurements. At these places, the second type of short cycle is missing a horizontal edge, because so far we are not assuming that we know anything about the state before the first round or after the last round. Therefore, for example, we cannot distinguish between two measurement errors in the first round and a qubit error just after the first round, or two measurement errors in the last round and a qubit error just before the last round.

This problem actually reflects a difficulty with fault-tolerant computation that we have mentioned back in Section 1: Encoding a physical qubit (in an arbitrary state) or decoding back into a physical qubit cannot be made fault tolerant. If we want our 3D decoding procedure to fully make sense, then we need to consider more carefully how we want to begin and end the computation.

**Fault-tolerant logical measurement and initialization.** To fix the time boundary problems, what we need is a way to make the first round and the last round of stabilizer measurements *perfect*. If we could do that, then the decoding graph no longer needs to include vertical boundary edges for these measurement rounds (because edges only represent errors), and the problematic short cycles will disappear.

Surprisingly, the last-round problem almost solves itself when we perform a destructive logical *Z* measurement. Recall that, to perform a destructive logical measurement of a Pauli operator, we simply need to measure each of its single-qubit factors individually. Since the surface code has multiple *Z* logical operator representatives, we want to measure *all* of them, which is easy due to the CSS structure of the surface code: we just measure every physical qubit in the *Z* basis.

But when we measure every physical qubit in the *Z* basis, we also incidentally measure all the stabilizers. Of course, these measurement results are not perfect in the literal sense: measuring data qubits is subject to the same measurement error as measuring ancilla qubits, and combining multiple measurement results will only make the error rate higher. However, these errors do not “behave like measurement errors”: A measurement error on a data qubit will flip both stabilizer results derived from that measurement, so these errors always appear in pairs except on boundaries. In fact, an imperfect measurement is equivalent to a stochastic Pauli error plus a perfect measurement, so in this sense we can “move” all errors before the measurement and claim that these *Z* stabilizer measurements are indeed perfect.

One catch is that we only measure the *Z* stabilizers this way and in fact, completely lose all *X* stabilizer information altogether. However, it turns out that this is not that important, since when using the MWPM decoder, *X* stabilizer information is only used for decoding *Z* errors, but when we perform a logical *Z* measurement, we no longer care about *Z* errors since the *X* logical information is destroyed anyway. The perfect *Z* stabilizer measurements are enough to fault-tolerantly decode *X* errors, the type of error that can affect the logical measurement result. Of course, the same method can also be used to implement a logical *X* measurement, with the roles of *Z* and *X* swapped.

The same idea can actually also be used at the beginning of a circuit. For example, we can initialize all physical qubits to |0〉 . In this state, we have “perfect” knowledge of all *Z* stabilizers as well as the *Z* logical operator, but no knowledge of any *X* stabilizers—they are actually still in quantum superposition. Then we start the repeated stabilizer measurements. After the first round of *X* stabilizer measurements, the state approximately (“Approximately” is the best we could hope for when initializing a state with imperfect operations.) collapses into a surface code state, but with random initial *X* stabilizer values, it is like every qubit was subject to a *Z* error with 50% probability. Conveniently, we do not care about these *Z* errors because we are initializing the logical state to $|\bar{0}\rangle$ , which is invariant under a logical *Z* error anyway. To prepare the $|\bar{1}\rangle$ state, we can “pre-apply” a logical *X* gate by initializing a column of qubits to |1〉 instead (For the d×d surface code with an odd *d*, we can also initialize all qubits to |1〉 , since the entire qubit grid is the disjoint union of an odd number of columns.). Symmetrically, we can also initialize the logical state to $|\bar{+}\rangle$ or $|\bar{-}\rangle$ .

There is an interesting point worth considering: If we initialize each qubit arbitrarily to 
|0〉
 or 
|1〉
, such that each row does not necessarily have the same parity, are we preparing a logical 
$|\bar{0}\rangle$
 or 
$|\bar{1}\rangle$
? The answer is that the logical *Z* operator representative we are using matters here, because different logical *Z* operator representatives differ by some *Z* stabilizer generators, and when we initialize each qubit arbitrarily, some of the “perfect” *Z* stabilizers will have value 
−1
.

Combining our fault-tolerant logical initialization and measurement procedures gives the *quantum memory experiment*, one of the simplest fault-tolerant quantum computation experiments that might be somewhat boring but is not trivial: First, initialize the logical qubit to $|\bar{0}\rangle$ , $|\bar{1}\rangle$ , $|\bar{+}\rangle$ , or $|\bar{-}\rangle$ . Then repeat stabilizer measurements for any number of rounds, and finally measure the logical qubit in the same basis it was initialized in. The experiment is not trivial because, if only detector information is given to the decoder, then the decoder has no way of knowing whether the state was initialized to $|\bar{0}\rangle$ or $|\bar{1}\rangle$ , or knowing whether, in the final measurements, an entire column of measurement results is flipped. Therefore, even if the correct measurement result is obvious “from the outside”, the decoder has to actually reason about the errors that may have occurred to output a set of corrections that will lead to this result.

There have been many attempts to perform the quantum memory experiment on real hardware; the latest one, reported in [13], has successfully made the lifetime of the logical qubit longer than its best physical qubit, though only by a factor of 2.4 . There have also been more simulations of the quantum memory experiment, testing various decoders, code variants, error models, etc. The reason that people care about the memory experiment is less that preserving the logical qubit is in itself important and more that the identity gate (i.e., idling) is the simplest quantum gate, and the accumulation of errors would always be a problem for useful quantum computation consisting of more than a few thousand quantum gates. In fact, as we will see in Section 5.2, many logical operations can be implemented with circuits very similar to that of the memory experiment.

**Circuit-level error model.** Before we discuss logical operations, we want to rectify the issue of the error model. We have been using the phenomenological error model, which allows us to ignore the details of the stabilizer measurements. However, to convincingly argue that fault-tolerant quantum computation is indeed possible, the error model needs to at least take into account that every component of the quantum circuit can be faulty, including initialization, measurement, and all gates. Fortunately, as we will see, a complete circuit-level error model is not qualitatively different from the phenomenological error model as far as the *existence* of a threshold is concerned.

**Figure 5:**
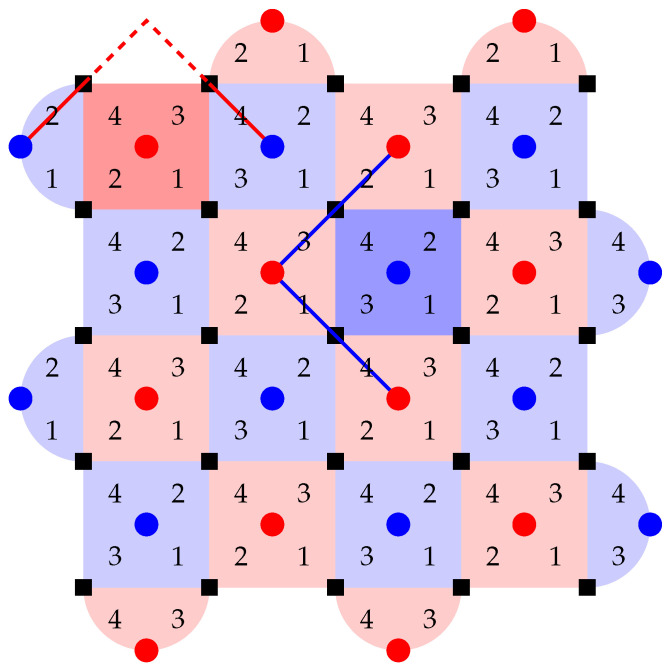
A valid stabilizer measurement schedule for the surface code that avoids “bad” hook errors, with examples of two hook errors.

We first concretely specify some details of our stabilizer measuring circuit. We must at least perform $O(d^{2})$ stabilizer measurements in parallel so that the circuit depth for an entire round remains constant and idle errors accumulated within each round do not scale up with *d*. In fact, with some careful scheduling, it is possible to perform *all* stabilizer measurements in parallel, within 4 layers of CNOT gates. One possible pattern is shown in Figure 5. The requirements for a schedule to be valid are analyzed well in [14]:

- The same qubit cannot be used twice in the same CNOT layer. This is naturally satisfied if, in each layer, all CNOT gates have the same direction from the perspective of the ancilla qubit (for example, in layer 1, every ancilla qubit interacts with the adjacent data qubit on its southeast (if any), so every data qubit interacts with the ancilla qubit on its northwest (if any)). In the schedule in Figure 5, the CNOT direction is different between *Z* and *X* ancilla in layers 2 and 3, but they are both in the northeast–southwest direction, and ancilla qubits only see the same type of ancilla qubits in a diagonal direction, so as long as the direction is consistent between the same type of ancilla qubits, this will not cause any conflict.
- When two stabilizers of different types overlap on two qubits, an important pitfall to avoid is the pattern below:(14)
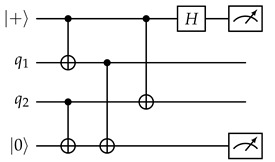
  Here, there is a CNOT chain from the *X* ancilla qubit (initialized to |+〉 ) the *Z* ancilla qubit (initialized to |0〉 ) through $q_{1}$ , causing the measurement result of the latter to become random (The measurement result of the *X* ancilla also becomes random due to a reverse CNOT chain from the *Z* ancilla to the *X* ancilla.). But the following two patterns are both fine:(15)
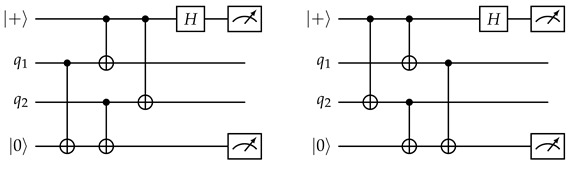
  In the first diagram, due to the ordering of CNOT gates on each data qubit, there is no CNOT chain from the *X* ancilla to the *Z* ancilla. In the second diagram, there are two such CNOT chains, which cancel each other and do not affect the measurement result.
  Therefore, the rule is: For each pair of data qubits $q_{1}$ and $q_{2}$ adjacent to the same pair of ancilla qubits $q_{x}$ and $q_{z}$ , they must interact with those ancilla qubits in the same order, i.e., if $q_{1}$ – $q_{z}$ comes before $q_{1}$ – $q_{x}$ , then $q_{2}$ – $q_{z}$ also comes before $q_{2}$ – $q_{x}$ , and vice versa. The schedule in Figure 5 satisfies this constraint because both $q_{1}$ and $q_{2}$ will always interact with $q_{x}$ and $q_{z}$ in reading order: either left before right or top before bottom.
- For each *Z* ancilla qubit, we want the last two data qubits interacted with to be vertically adjacent, and for each *X* ancilla qubit, we want them to be horizontally adjacent. This constraint is related to the direction of logical operators, and it serves to suppress *hook errors*, which will be described below. This is exactly the reason why the two ancilla types need to have different CNOT directions in layers 2 and 3.

Having fixed the stabilizer measurement circuit, we will now look at the circuit-level error model. We still model all errors as stochastic Pauli gates: an error on a unitary gate is always equivalent to the ideal unitary gate followed by an error channel, and we approximate that error channel as a stochastic Pauli gate. For the CNOT gate, we allow the Pauli errors applied to its two qubits to be correlated, but this is not that important after decomposing the error to *Z* and *X* components, since an XX error after the CNOT gate is equivalent to an XI error before it, and a ZZ error after the CNOT gate is equivalent to an IZ error before it. Therefore, after decomposing, each individual error is equivalent to a single-qubit *Z* or *X* Pauli somewhere between circuit components.

We now analyze all possible locations for such a single-qubit Pauli error. By symmetry, we can explicitly analyze just *X* errors:

- If an *X* error occurs on a data qubit, the *X* error will commute with CNOT gates between the data qubit and *X* ancilla qubits (because such CNOT gates have the data qubit as the target), so we only need to consider its location relative to the (up to) two CNOT gates between the data qubit and *Z* ancilla qubits.
  - If it is before the first such gate or after the second one, then it is equivalent to an *X* error between two syndrome extraction rounds, which is already handled in the phenomenological error model as a horizontal edge.
  - If it is between these two gates, then in this measurement round it will only flip the second *Z* stabilizer, but starting from the next round it will flip both stabilizers. This is equivalent to the combination of a horizontal edge and a vertical edge, but since it is a single error, in order to weight error configurations correctly, we need to add it into the decoding graph as a single edge. This type of edge is known as a *diagonal edge*.
- If an *X* error occurs on a *Z* ancilla qubit, then the error will not propagate to data qubits, but it will cause the measurement result of that ancilla qubit to be flipped (only for this measurement round). This is already handled in the phenomenological error model as a vertical edge.
- If an *X* error occurs on an *X* ancilla qubit, it will not affect the measurement result of that ancilla qubit, but it will propagate to all adjacent data qubits that interact later with this *X* ancilla qubit in this round. Depending on the temporal location of the error, up to 4 data qubits may be affected, but many of these cases can be simplified:
  - Propagating to all 4 qubits is equivalent to a no-op, since XXXX on these 4 qubits is a stabilizer, and scheduling constraints ensure that adjacent *Z* stabilizers see either zero or two *X* during this round. (This should be expected, as an *X* error on a qubit just initialized to |+〉 should not affect anything!)
  - Propagating to the last 3 qubits is equivalent to propagating to the first 1 qubit only, which is in turn equivalent to a single data qubit error before this round.
  - Propagating to the last 1 qubit is equivalent to a single data qubit error after this round.
  - Only propagating to the last 2 qubits results in a new type of error, the *hook error*, with some examples shown in Figure 5. Thanks to our third scheduling constraint stated previously, the direction of a hook error is always perpendicular to the logical operator of the same type. In the schedule in Figure 5, it is always the case that one of the detectors affected by the hook error sees it in this round and the other detector only sees it in the next round, so the corresponding *hook edge* is the combination of three edges, two horizontal and one vertical.

In summary, we are able to modify the 3D decoding graph for the phenomenological error model to adapt to the circuit-level error model by adding diagonal edges and hook edges. In the new decoding graph, any path of *X* errors that goes from the top boundary of the code to the bottom boundary still must have at least length *d*, thanks to *X* hook errors not moving in this direction. The degree of the graph also remains constant, albeit a larger constant (12 instead of 6). Therefore the proof of threshold outlined at the end of Section 4 still applies, just with some constants changed. In conclusion, the surface code memory experiment has a fault-tolerant threshold under our circuit-level error model.

**Defect diagram.** Drawing the 3D circuit-level decoding graph explicitly would be a little too messy (the reader can refer to [10] for an example). Now that we have looked at some gritty details and have a sense of how everything works out, we want to switch to a more abstract representation for developing methods to implement logical operations.

**Figure 6:**
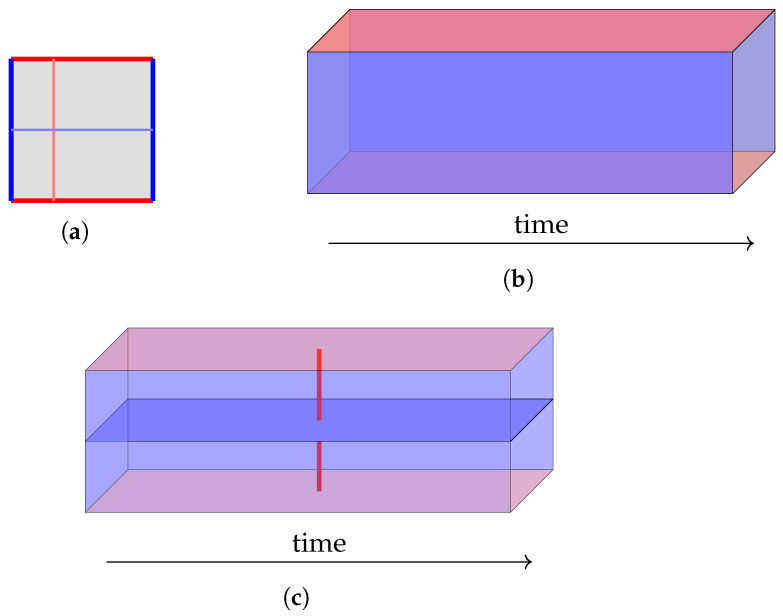
(**a**) The defect diagram of a qubit and two example logical operators. Thick blue and red border lines represent code boundaries with additional *Z* and *X* boundary stabilizers, respectively. These boundary stabilizers prevent Pauli strings of the *opposite* color to “escape” from these boundaries. Thin blue and red internal lines represent *Z* and *X* logical operators. (**b**) The defect diagram of the same qubit in 3D. The top and bottom walls are red, and the front and back walls are blue. The 2D defect diagram in (**a**) can be regarded as a space-like cross section of this 3D diagram. The left and right ends of this “tube” are drawn in gray, indicating that what happens at the time boundaries is not yet specified. (**c**) The defect diagram of a *Z*-basis memory experiment. The left and right ends are now blue walls. The blue internal surface is a logical membrane corresponding to a *Z* logical *observable*, and the red line is a logical string corresponding to an *X* logical *operation*.

We note that the most important feature of a decoding graph is its *boundaries*. In the 2D “static” decoding graph of the surface code, *Z* strings are only allowed to “escape” from the left and right boundaries, and *X* strings only from the top and bottom boundaries. This decides the directions of the logical operators. We draw a simple *defect diagram* (The name “defect diagram” is used in [15]; here “defect” refers to locations where the properties of the bulk of the code—*Z* parity and *X* parity conservation—are broken, such as boundaries and twists.) to represent this information in Figure 6a. Note, again, that a logical operator always connects two disjoint boundary segments of the same color (Actually, this is only true because the bulk region is simply connected. A bulk region with holes would allow a more complicated topology.); strings connecting the same boundary segment are stabilizers. Therefore, the minimum distance between different boundary segments of the same color represents the code distance (One catch is that, since the defect diagram does not represent details such as the direction of the hook error, distances seen on the defect diagram is only a rough approximation of distances on the decoding graph.).

When we extend this idea to the 3D decoding graph, the qubit becomes a “tube” in the time direction, as depicted in Figure 6b (The author prefers to draw the time axis going from left to right, because in the vertical direction it is hard to say whether it is more intuitive for time to go from bottom to top or from top to bottom!). Each 1D boundary of the original surface code becomes a 2D “wall” of the tube, and they still only let the correct color of strings escape. We do not yet “cap” the left and right ends of the tube, so this diagram simply represents the preservation of a qubit, leaving it unspecified what we are doing at the beginning and end of this time period.

Finally, we draw a slightly different defect diagram in Figure 6c that represents a *Z*-basis memory experiment, meaning that we specify that the qubit is initialized to $|\bar{0}\rangle$ or $|\bar{1}\rangle$ and measured in the *Z* basis. Importantly, both our initialization and measurement correspond to blue *time boundary* walls: Due to the perfect *Z* stabilizers, *X* measurement errors (which only affect *Z* stabilizers) can never escape from the time boundaries, but *Z* measurement errors (affecting *X* stabilizers) can escape without problem.

When we add these blue time boundary walls, something interesting happens: all blue walls in the defect diagram become a connected segment, while the red walls are still separate. Therefore, this diagram only has red logical operator strings, not blue ones. This reflects the fact that only *X* logical errors can meaningfully change the semantics of this simple circuit, and thus only *X* logical errors can be corrected. The distance between the red walls is the fault-tolerant distance of the memory experiment.

But what about the *Z* logical operator that we are measuring in the end? It turns out that, when studying the 3D decoding graph of a fault-tolerant procedure, one needs to differentiate between logical operators as *operations* and logical operators as *observables*. A logical operation can be performed at any single time point, so it corresponds to a 1D string as before. A logical observable, on the other hand, is something we try to preserve over time, so it should be a 2D *membrane* that spans the diagram in the time direction. Both types of logical operators are shown in Figure 6c.

Operationally, the logical membrane provides an elegant method for determining the result of a logical measurement from a set of errors. Every *X* error that *crosses* the *Z* logical membrane is an *X* error that flips a qubit on the *Z* logical operator, so whether the set of errors crosses the membrane an odd or even number of times determines whether the logical measurement result is flipped. One can verify from the defected diagram that here a logical operator string always crosses the membrane an odd number of times, and “stabilizer-like” short cycles always an even number of times.

### 5.2. Lattice Surgery

*Lattice surgery* [12] is a method to implement fault-tolerant logical operation by merging and splitting surface code lattices. Compared to its predecessor, defect braiding [16], it can directly work on d×d surface code patches, thus incurring much less qubit overhead, while preserving the advantage of only requiring local connectivity. Technically, lattice surgery can only directly implement Clifford gates, but it combines well with *state injection*, which allows initializing logical qubits to an arbitrary *noisy* state. For a certain class of non-stabilizer states known as *magic states*, noise can be *distilled* away with many copies and only Clifford gates, eventually allowing fault-tolerant implementation of a non-Clifford gate and thus universal quantum computation.

A good way to understand lattice surgery is through the defect diagrams of various lattice surgery procedures, which clarify both the logical operation implemented by each procedure and the constraints required for fault tolerance. In fact, the defect diagrams of many such procedures are quite similar to that of the memory experiment.

**Figure 7:**
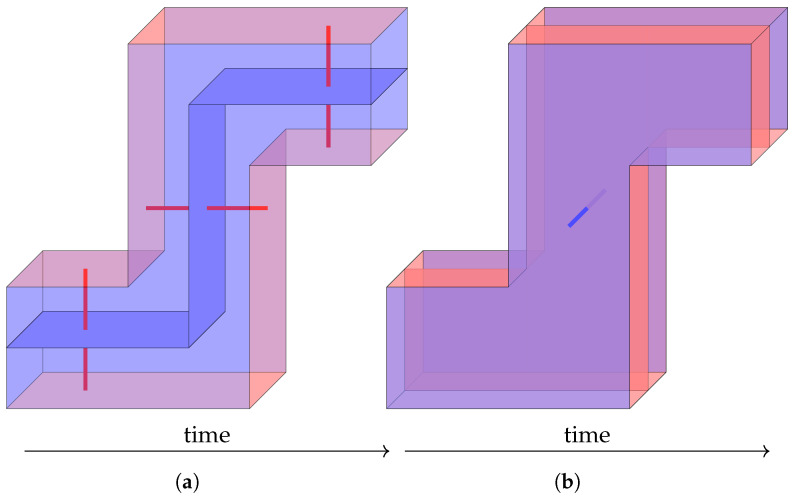
Defect diagrams of the (**a**) *Z*-basis and (**b**) *X*-basis memory experiments with a moving qubit. Both diagrams are in the shape of a bended tube, with the front and back sides blue and other sides red. The two diagrams differ in the colors of the left and right ends of the tube, which are blue in (**a**) and red in (**b**). Some relevant logical membranes and logical strings are also shown.

**Moving a qubit.** A commonly used method to manipulate defect diagrams is topological *deforming*. By deforming the defect diagram of the memory experiment, we get the defect diagrams shown in Figure 7, where a logical qubit is moved to another location in a large lattice of physical qubits. Figure 7a,b depict *Z*-basis and *X*-basis memory experiments using such a qubit, although it is relatively easy to convince oneself that such a qubit can be used anywhere in place of a normal qubit. It can be regarded as a module that can be used in more complicated lattice surgery procedures, with two “ports” to connect to other modules.

As abstract diagrams, Figure 7 is quite straightforward, but it does need a few points of explanation regarding circuit-level implementations. The diagram has four walls in the “time boundary” direction, two for qubit initialization and two for qubit measurement. The first initialization is for the logical qubit, and it is in the basis of the memory experiment, but the second initialization is always in the *X* basis and extends the existing square surface code patch to a longer rectangular one: all the new physical qubits are initialized to |+〉 . Note that if we knew all the *X* stabilizers of the existing surface code patch perfectly, then we would also know all the *X* stabilizers of the new surface code patch: each of them is either the same as an old stabilizer, or a set of qubits newly initialized in |+〉 , or a combination of both (at the boundary between the old patch and the new part). In reality, we do not exactly know the old stabilizers because of imperfect measurement, but those measurement errors just correspond to vertical edges inside the defect diagram. No *Z* error is allowed to “escape” through the new boundary, not even between it and the existing spatial boundary—the connection between walls of the same color is seamless.

Similarly, the first measurement time boundary (third time boundary overall) measures a subset of all the physical qubits in the rectangular surface code patch, shrinking it back into a square. This gives a “perfect” measurement result to all *X* stabilizers not in the square patch and also allows calculating some boundary stabilizers of the square patch from the corresponding bulk stabilizers of the rectangular patch. Note that among the *X* logical operators depicted in Figure 7a, one is in the time direction (i.e., consists of vertical edges) and goes from the “patch extending” time boundary to the “patch shrinking” time boundary. Therefore, to maintain the fault-tolerant distance *d*, these two lattice surgery operations must be at least *d* rounds apart, which will turn out to be an important constraint in many lattice surgery procedures.

We also need to explain the physical meanings of the logical observable membranes. In Figure 7a, the new ingredient is a *space-like* (perpendicular to the time direction) portion of the membrane. This represents the fact that, at some point during the move, we need to switch from one *Z* logical operator representative to another, which is achieved by multiplying by some stabilizer generators. Of course, in an experiment, we would not know the exact values of these stabilizers and would instead use imperfect stabilizer measurement results in some stabilizer measurement round. A measurement error during this round would flip the logical operator, but if that measurement error is caught by the decoder, this flip can be corrected. This also explains the time-like *X* logical operator in Figure 7a: since both relevant time boundaries are *X* time boundaries, we do not get perfect information about *Z* stabilizers, and if all *d* rounds of measurements on one stabilizer happen to be all flipped, we will multiply by an incorrect stabilizer value, the decoder will not catch it, and a logical error will occur.

In Figure 7b, the logical *X* membrane intersects with 4 separate time boundaries, instead of just twice at the beginning and the end. This means that the logical *X* observable also needs to be adjusted during the moving procedure: the static (1D) logical *X* operator of the rectangular patch will be longer than that of either square patch, so the *X* observable needs to be multiplied by the initial *X* values of some physical qubits (although the result is trivial if all these qubits are initialized to |+〉 ) when extending the patch and multiplied by some of the *X* measurement results when shrinking the patch. The need for these adjustments is exactly why these physical initializations and measurements must be in the *X* basis.

The tube in Figure 7 can in fact be bent in any way without changing the logical semantics, as long as opposite walls remain separated by at least distance *d*. Moving a logical qubit any distance, along any path, takes *d* rounds of stabilizer measurement. This does not violate the locality principle because any logical measurement result on the moved qubit is only meaningful after making the necessary adjustments, which amounts to transmission of some classical information. This situation is essentially a sort of quantum teleportation. In fact, we can even send qubits “backward in time” by bending the tube in an “S” shape.

**Multi-qubit logical measurement.** It is instructive to analyze what happens when we send a logical qubit through an S-shaped tube. The first part of the procedure is a C-shaped tube, which creates two logical qubits such that, when we measure both in the *Z* basis or both in the *X* basis, the results will be perfectly correlated. Indeed, these logical qubits are in a Bell state, i.e., one of

(16) $$\begin{array}{c} |\Phi^{+}\rangle =\frac{1}{\sqrt{2}}\left(|00\rangle +|11\rangle \right),\;|\Psi^{+}\rangle =\frac{1}{\sqrt{2}}\left(|01\rangle +|10\rangle \right),\\ |\Phi^{-}\rangle =\frac{1}{\sqrt{2}}\left(|00\rangle -|11\rangle \right),\;|\Psi^{-}\rangle =\frac{1}{\sqrt{2}}\left(|01\rangle -|10\rangle \right). \end{array}$$

which one exactly depends on the ZZ and XX observable values, which are determined by the “adjustment terms” obtained when performing the initial procedure.

Conversely, the second part of the S-shaped tube, a mirrored C-shaped tube, takes two logical qubits and performs a destructive Bell measurement, i.e., simultaneous measurements of ZZ and XX . The “adjustment terms” become the measurement results. Therefore, the S-shaped tube actually implements the most basic quantum teleportation protocol on the logical level.

**Figure 8:**
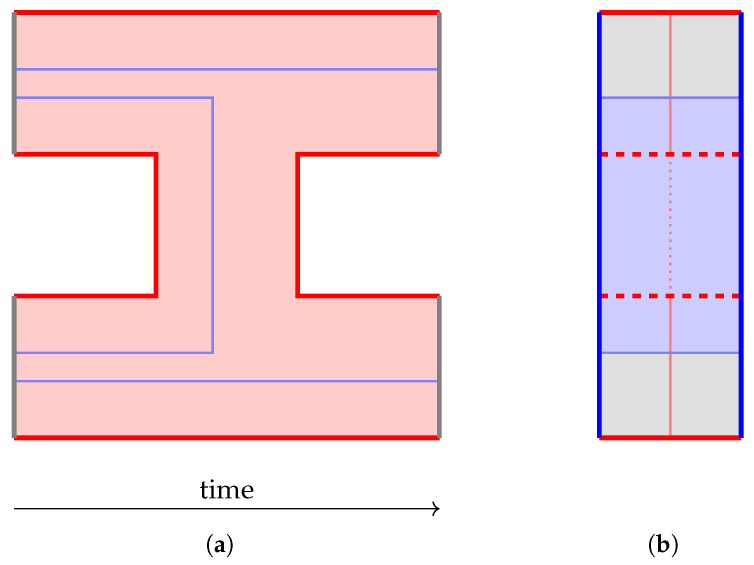
(**a**) A time-like cross section of the defect diagram of a non-destructive ZZ logical measurement. The unseen front and back walls are both blue. Gray lines on the left and right ends are unspecified “ports” that can be red or blue walls depending on the type of initialization/measurement, or they can be connected to other ports on other modules. The three internal blue lines are three *Z* logical membranes (A practical implementation of the decoding module would probably choose the logical representatives so that these membranes partially overlap, but here we separate them for clarity.): The bended one corresponds to the ZZ logical measurement yielding the correct result, and the straight ones correspond to logical *Z* observable of the first/second qubit being preserved. The entire cross section is also an *X* logical membrane, corresponding to the logical XX observable being preserved. (**b**) A space-like cross section of the same defect diagram, focusing on the moment of the two patches merging. The dashed thick red lines represent segments of red walls before the merge, and the dotted red line represents a segment of the *X* logical membrane after the merge.

This also gives us a hint on how to implement a *non-destructive* multi-qubit logical Pauli measurement. A non-destructive Pauli measurement procedure should, in addition to the classical measurement result, output the same number of qubits at the input, and all logical observables *commuting with the measured Pauli* should be preserved. For a ZZ logical measurement, this means the individual *Z* observables of each qubit (i.e., ZI and IZ ), and the joint XX observable. A concrete defect diagram is given in Figure 8. Here, two surface code patches are merged at some point, remain merged for at least *d* rounds, and then are split into two separate patches again. Indeed, merging and splitting are often referred to as the fundamental techniques of lattice surgery, and these techniques alone allow implementation of many interesting logical operations.

One way to demonstrate the power of merging and splitting is to note that the CNOT gate can be implemented with two-qubit ZZ and XX measurements and one logical ancilla qubit: (17)
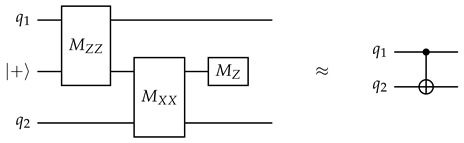
Here, the “≈” means that some Pauli corrections may need to be applied depending on the measurement results, which is equivalent to adjusting the values of certain logical observables. With CNOT gates, we can then implement non-destructive multi-qubit measurements involving any number of qubits (As long as it is an all-*Z* or all-*X* Pauli; for a Pauli operator with mixed terms, we also need Hadamard and/or *S* gates.).

**Figure 9:**
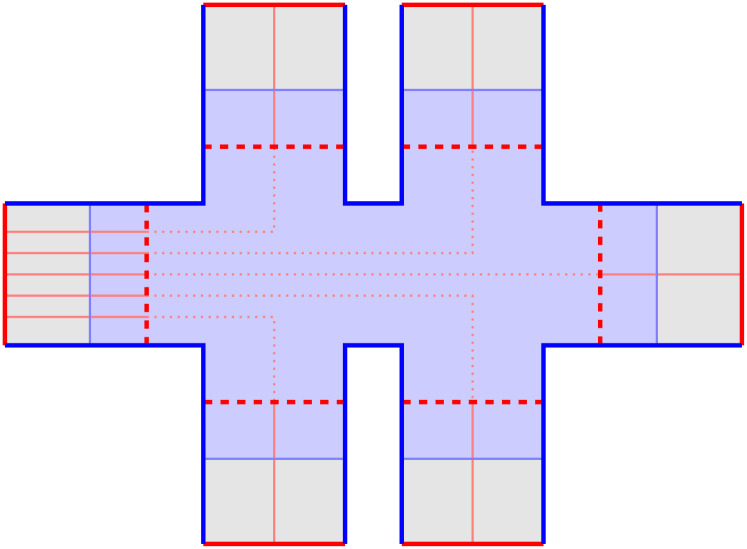
A space-like cross section of the defect diagram of a non-destructive ZZZZZZ logical measurement, with a view akin to Figure 8b.

However, ref. [12] describes a more efficient method for directly implementing Pauli measurements that involve more than two qubits. The general technique is demonstrated in Figure 9. We explicitly show how this procedure preserves $X_{1}X_{k}$ for k=2,3,4,5,6 ; other all-*X* logical operators with an even number of *X* can always be written as products of these five Pauli operators. Another interesting point is that the merged patch has 12 boundary segments, 6 red and 6 blue. Such a patch can store 6−1=5 independent logical qubits, which indeed matches the size of the Hilbert space of 6 qubits after a single projective measurement.

**Arbitrary Clifford circuits.** With merging and splitting essentially equivalent to the power of CNOT gates, we still need Hadamard gates and *S* gates for implementing an arbitrary Clifford circuit. The Hadamard gate is relatively easy: one can apply the Hamamard gate *transversally* to a surface code patch, i.e., apply it to every physical qubit. The resulting state will be encoded in a new surface code, where all *Z* stabilizers become *X* stabilizers and vice versa (If implemented on a large lattice of physical qubit, this will flip the *parity* of the surface code grid, which can be fixed via physical SWAP gates like in [16], or elongated stabilizers like in [17,18].), and the *Z* and *X* logical operators are also swapped. In the defect diagram, such a transversal Hadamard gate is a *domain wall* that cuts through the bulk, such that everything red that passes through it becomes blue and vice versa, including boundaries, logical strings, and logical membranes. This does not actually change the structure of the decoding graph locally and will only matter when the defect diagram contains non-trivial loops (e.g., when initializing two qubits to a Bell state, applying a Hadamard gate to only one of them, then performing a logical ZZ measurement), where the *Z* decoding graph will loop back and connect to the *X* decoding graph. Note that a transversal Hadamard gate creates a space-like domain wall, but time-like domain walls can also be easily created by swapping *Z* and *X* in only half of a surface code patch, causing the stabilizers on the domain wall to become ZZXX stabilizers.

The *S* gate (the π/2 phase gate) is somewhat more difficult to implement. It is equivalent to the Pauli *Y* measurement, which requires measuring the *Z* and *X* logical operators of the *same* surface code patch together, whereas a simple domain wall will only allow measuring *Z* and *X* together on different qubits. The earliest scheme [16] actually implements the *S* gate in a similar way to the *T* gate (the π/4 phase gate), with distilled magic states, which are associated with significant spacetime overheads. The scheme in [17] improves upon this by allowing the “magic state” to be indefinitely reused, with the key observation that any Pauli operator with an even number of *Y* can be measured easily (e.g., to measure YY , one can initialize a third logical qubit to |+〉 , and then measure ZZZ , XXZ , and IIX in this order), and a single logical qubit stabilized by *Y* can help change the number of *Y* in any Pauli operator from odd to even. This allows the high cost of magic state distillation to be incurred only once, making it more acceptable.

Meanwhile, Refs. [12,14] deals with the *Y* measurement problem by introducing a new component to the surface code, a *twist*. Topologically, it is what happens when a loop in a defect diagram that crosses a domain wall an odd number of times is shrunk to a single point. On the circuit level, it can be implemented with a weight-5 stabilizer with a *Y* component, which does lead to complications compared to normal surface code stabilizers. A method for dealing with these complications is proposed in [18], with the observation that an “invalid” *tangled* schedule for measuring two stabilizers can be modified slightly to instead measure the *product* of those two stabilizers, which gives a way to measure the elongated twist stabilizer without changing the connectivity requirement for the hardware. Another even more efficient method of *Y* measurement is designed in [15], by carefully topologically deforming a defect diagram with a twist to reduce its volume while maintaining a fault-tolerant distance of at least *d*. It is implemented with some complicated physical gate patterns that are nevertheless compatible with the normal surface code connectivity.

In summary, Hadamard and *S* gates add some complexity, but there exist schemes to handle them relatively cheaply and within the broad framework of lattice surgery. In contrast, currently known schemes to implement non-Clifford gates require another layer of errors and error correction on top of lattice surgery.

### 5.3. Non-Clifford Gates via Magic States

**State injection.** Executing stabilizer circuits with lattice surgery is ultimately like a glorified memory experiment. The procedure for tracking error corrections is essentially the same as the procedure for simulating the circuit with the Gottesman–Knill theorem, and in the end we give the decoder a quiz that we already know the answer to. Such experiments may be good for benchmarking the quantum hardware and the decoder, but the quantum computer is not contributing anything computationally.

However, for a circuit to be a stabilizer circuit, not only must all its gates be Clifford gates and all its measurements be Pauli measurements, but also all its qubits must be initialized in stabilizer states. Allowing qubit initialization into arbitrary states will open up a new world of possibilities, eventually leading to universal quantum computation.

There are in fact simple procedures, known as *state injection*, to encode an arbitrary physical qubit into a logical surface code qubit, with the mandatory catch that they are not fault-tolerant. However, even noisy non-stabilizer states allow us to jump out of the scope of the Gottesman–Knill theorem, and with many copies of them, we can try to reduce the noise with the fault-tolerant Clifford operations allowed by lattice surgery, distilling the “magic” from these noisy states.

**Figure 10:**
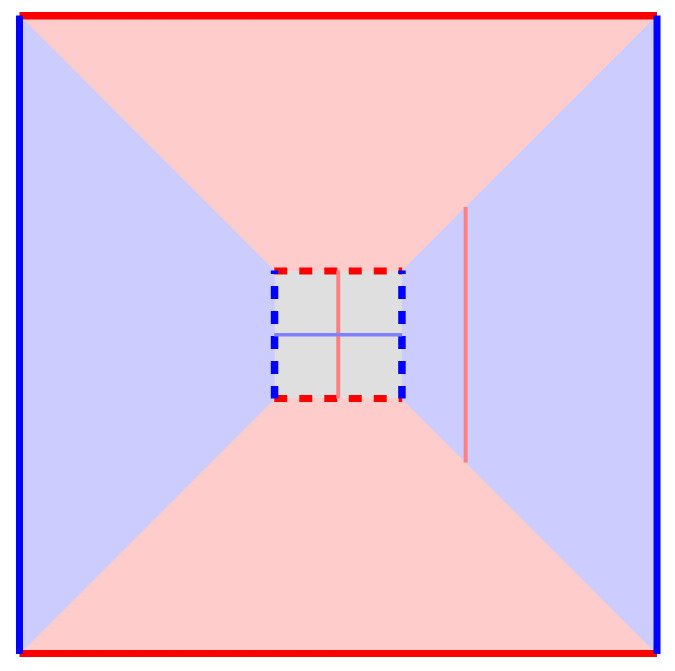
A space-like view of the defect diagram of a state injection procedure. All thinner lines are logical operator strings; two of them are of length 1, and the other is longer but still shorter than the distance *d* of the final code.

Figure 10 shows the defect diagram of a state injection procedure. The procedure can be carried out in a single stabilizer measurement round: We place the physical qubit to be injected at the center of the patch, initialize all other physical qubits in the patch to either |0〉 or |+〉 following the blue and red shades in the defect diagram, and measure all the stabilizers of the patch. This procedure is evidently not fault-tolerant: there are not only length-1 logical operators corresponding to errors occurring directly on the input physical qubit at the very start, but also other short logical operator strings near the point of injection. Therefore, the logical error rate would not decrease when the code distance *d* increases. However, as long as the physical error rate *p* is below the threshold, the logical error rate should also not increase all the way to 0.5 , but should be upper bounded by a constant value scaling as O(p) when p→0 , which is all we need for a state injection procedure.

**Gate teleportation.** A non-stabilizer state can be converted to a non-Clifford gate through *gate teleportation*. Below is an example circuit that uses the non-stabilizer state T|+〉 to implement the *T* gate: (18)
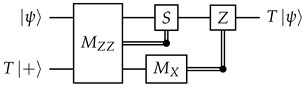
Here, the measurement results of $M_{ZZ}$ and $M_{X}$ are used as control bits for the classically controlled *S* and *Z* gates, respectively. The fact that this circuit is equivalent to a simple *T* gate can be seen as follows:

- Inputting the magic state T|+〉 is equivalent to inputting |+〉 and then applying a *T* gate. Furthermore, the *T* gate commutes with the $M_{ZZ}$ . Therefore the original circuit can be transformed to:(19)
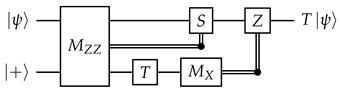
- Depending on the measurement result of $M_{ZZ}$ , the joint state of both qubits is projected to either Span(|00〉,|11〉) or Span(|01〉,|10〉) . In the first (resp. second) subspace, the *T* gate on the lower wire is equivalent to a *T* gate (resp. a $T^{†}$ gate) on the upper wire. Therefore the circuit is further transformed into:(20)
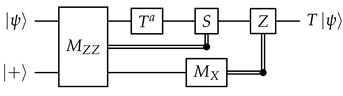

where a=±1 represents the measurement result of $M_{ZZ}$ .
- Since the *S* gate is classically controlled by *a*, it corrects the $T^{a}$ gate to an unconditional *T* gate. The *T* gate can then be commuted back through the $M_{ZZ}$ , resulting in:(21)
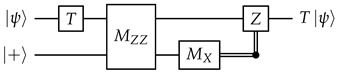
- Now note that the side effect of $M_{ZZ}$ is equivalent to applying the Pauli ZZ gate with probability 50% . Since the ancilla qubit is initialized to |+〉 , the result of $M_{X}$ reflects whether the ZZ gate has been applied, and the controlled *Z* gate corrects it on the upper wire if it has been. Therefore the latter part of the circuit above is equivalent to an identity gate, and the entire circuit is equivalent to:(22)
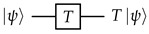

Therefore, the circuit (18) indeed implements the *T* gate.

We note that the classical controlled *Z* gate can be implemented simply by adjusting the values of logical operators that anti-commute with this *Z*, without applying any additional physical operation. The classical controlled *S*, however, must be applied physically, either as an explicit lattice surgery procedure or by switching between an *X* measurement and a *Y* measurement sometime in the future. Either way, this requires the ability to obtain error-corrected logical measurement results without having access to the full decoding graph, which is possible by treating edges connecting to unknown regions of the decoding graph as boundary edges. As long as the cutoff is far enough from the relevant logical operators, the fault-tolerant distance will be retained.

Of course, the fidelity of a gate implemented through gate teleportation is dependent on the fidelity of the input *magic state* T|+〉 . With a noisy magic state created directly by state injection, the resulting *T* gate will be noisy too. However, if this is the only non-negligible source of noise, then it becomes much easier to implement a fault-tolerant *T* gate from these faulty ones.

**Magic state distillation.** The simplest magic state distillation protocol is a 15-to-1 protocol using a 15-qubit *(quantum) Reed–Muller code*. The 15-qubit Reed–Muller code is a CSS code with four weight-8 *X* stabilizer generators:

(23) $$\begin{array}{cc} X_{1}X_{3}X_{5}X_{7}X_{9}X_{11}X_{13}X_{15},\; & X_{2}X_{3}X_{6}X_{7}X_{10}X_{11}X_{14}X_{15},\\ X_{4}X_{5}X_{6}X_{7}X_{12}X_{13}X_{14}X_{15},\; & X_{8}X_{9}X_{10}X_{11}X_{12}X_{13}X_{14}X_{15}. \end{array}$$

Note how each *X* stabilizer generator corresponds to a binary bit in the physical qubit index. Among the set of *Z* Pauli operators that commute with all these *X* stabilizers, all even-weight ones are *Z* stabilizers, and all odd-weight ones are *Z* logical operator representatives. In particular, the set of weight-3 *Z* logical operators are:

(24) $$\{Z_{i}Z_{j}Z_{k}|1\leq i,j,k\leq 15,\;i⊕j=k\}.$$

The code distance of the 15-qubit Reed–Muller code is 3, meaning that it is capable of either correcting an arbitrary 1-qubit error, or detecting an arbitrary 2-qubit error.

For the purpose of implementing the *T* gate, an important property of the 15-qubit Reed–Muller code is that, when we measure every physical qubit in the *Z* basis, the number of |1〉 results will always be either 0 or 8 for the logical state $|\bar{0}\rangle$ , and either 7 or 15 for the logical state $|\bar{1}\rangle$ . For example, suppose we measure the first physical qubit $q_{1}$ of $|\bar{0}\rangle$ and obtain a result of |1〉 . Since $Z_{1}Z_{2}Z_{3}$ is a logical operator (with the value +1 for $|\bar{0}\rangle$ ), this means measuring $q_{2}$ and $q_{3}$ must give opposite results. But this is also true for $q_{4}$ and $q_{5}$ , $q_{6}$ and $q_{7}$ ... Therefore, there will be seven |0〉 results and seven |1〉 results among qubits $q_{2}$ to $q_{15}$ , and together with the initial |1〉 for $q_{1}$ , the number of |1〉 results will be 8 in total. This argument actually works no matter on which qubit $q_{i}$ we get the initial |1〉 —the other qubits can always be grouped into seven pairs of $q_{j}$ and $q_{i⊕j}$ . Therefore, either we never get |1〉 or we get 8 of them. The same is true with the logical state $|\bar{1}\rangle$ and the physical measurement result |0〉 .

The result above implies that $|\bar{0}\rangle$ (resp. $|\bar{1}\rangle$ ) is a superposition of computational basis states with Hamming weight 0 or 8 (resp. 7 or 15). Therefore, the transversal gate $T^{⊗15}$ (i.e., applying *T* on all 15 physical qubits) does not affect $|\bar{0}\rangle$ , but multiplies $|\bar{1}\rangle$ by a phase factor of $e^{7i\pi /4}=e^{-i\pi /4}$ , which means that it implements the logical $T^{†}$ gate.

With the help of this transversal $T^{†}$ gate, we can describe a protocol that converts 15 independent noisy instances of the T|+〉 state encoded in the surface code to a single $T^{†}|+\rangle$ state (still encoded in the surface code) with better fidelity:

1. Encode $|\bar{+}\rangle$ into a 15-qubit Reed–Muller code on top of the surface code.
2. Using gate teleportation, apply a *T* gate to each of the 15 “physical qubits” of the Reed–Muller code (logical qubits of the surface code), with the 15 noisy T|+〉 states as magic states.
3. Decode the resulting 15-qubit state into a single logical qubit of the surface code.

Steps 1 and 3 can be performed with Clifford circuits, so we assume that we have suppressed their error rates to a negligible level through lattice surgery. In step 2, an error on any input magic state will be directly translated to an error on the output state, i.e., a physical qubit of the Reed–Muller code. Therefore, if there are errors on at most 2 of the input magic states, those errors can be detected in step 3. We can try to correct the error, but that runs the risk of “correcting” a 2-qubit error in the wrong way, so a (usually) better approach is to simply declare failure and try again with freshly injected T|+〉 states. Assuming that an error occurs on each input magic state with probability *p*, there will be a probability of at least 1−15p that no error actually occurs, and we can declare success. We will also incorrectly declare success if there were an undetectable logical error in the Reed–Muller code, but that only happens with probability $O(p^{3})$ . As long as the initial *p* is low enough, the output state will have a lower error rate than the input states, conditioned on the procedure being declared successful.

To suppress the error rate of the magic state to an arbitrarily low level, we can chain multiple levels of the distillation procedure (The difference between T|+〉 and $T^{†}|+\rangle$ is trivial, fixable by an *S* gate which can be absorbed into the conditional *S* correction required for gate teleportation (meaning that when we would normally apply no correction, we apply an *S* gate, but when we would normally apply an *S* gate, we instead apply a cheaper *Z* gate, so that we still only apply the *S* gate half of the time).). The amount of resource needed scales exponentially with the number of levels: considering the cost of retrying, each level needs b=15/(1−15p) magic states from the previous level to produce a better magic state, so the amount of resource needed to produce a level-*k* magic state is $O(b^{k})$ . However, the error rate is suppressed *doubly* exponentially ( $O\left(p\right)\to O\left(p^{3}\right)\to O\left(p^{9}\right)\cdots$ ), so this amount of resource still grows only poly-logarithmically in the inverse of the target logical error rate, i.e., in the size of the logical circuit we want to execute.

Putting everything together, we now have a complete scheme for fault-tolerant universal quantum computation based on surface code and lattice surgery. An arbitrary quantum circuit can be approximately compiled into a Clifford+*T* circuit (In fact, many quantum circuits for interesting quantum algorithms can be compiled into Clifford gates and the Toffoli gate, which has an exact Clifford+*T* implementation.). Clifford gates can be directly implemented with lattice surgery. For each *T* gate, we inject many magic states T|+〉 , distill them into a single magic state with high enough fidelity, and then implement the *T* gate with gate teleportation. The overhead factor incurred by this scheme is poly-logarithmically bounded in theory. Of course, for practical implementation of this scheme, there are still many details that could be improved. We will look at some of these details in Section 6.

## 6. Research Problems

### 6.1. Physical-Level Implementation

**Hook error suppression.** In the sections above, when we switched to the defect diagram representation of lattice surgery procedures, we started to ignore the direction of hook errors. This is problematic because in many lattice surgery procedures (such as in Figure 9), the direction of short logical operators is not fixed for each Pauli type. For the purpose of demonstrating the theoretical result, this is acceptable, since we could always enlarge the patch by 2× wherever hook errors may halve the fault-tolerant distance. However, with the current cost of quantum hardware and various difficulties associated with scaling up, even a 2× qubit overhead is very significant, and thus it is worthwhile to find more efficient ways to handle hook errors.

In [14], a stabilizer measurement schedule is given that can switch the direction of the hook error between different code regions, allowing it to adapt to any possible boundary conditions in lattice surgery. However, this schedule takes 5 rounds of CNOT gates per stabilizer measurement round instead of 4. In contrast, Ref. [18] proposes an architecture that embraces the 2× qubit overhead by using the *unrotated surface code*, where the code boundary is set so that horizontal edges in the decoding graph run either parallel or perpendicular to the spatial boundaries, and hook edges run diagonally, which prevents hook errors from decreasing the fault-tolerant distance and allows for a more uniform stabilizer measurement schedule.

**Fabrication defects.** In superconducting quantum hardware, each qubit is a hardware component manufactured individually, and they all have slightly different parameters. Even after careful calibration, some qubits will perform worse than others, sometimes so much that it becomes *defective*, i.e., we are better off not using them than trying to endure their high physical error rates. The more qubits there are on a superconducting quantum chip, the more defective qubits there will be on average, and the less likely it becomes to avoid them altogether. Defective qubits disrupt the grid connectivity required by surface codes, demanding a modified surface code scheme that could work around them.

One technique for working around fabrication defects is *superstabilizers* [19,20,21], which locally modifies stabilizers near the site of the fabrication defect, potentially sacrificing a little code distance but not changing the overall topology of the code. Alternatively, when the defective qubit happens to be an ancilla qubit, one can use SWAP gates to change the roles of qubits near the defect site, still measuring all the original stabilizers, albeit at a reduced frequency. This approach can be very efficient if the hardware supports iSWAP gates, which allows combining SWAP gates needed for routing with CNOT gates already in the circuit, as demonstrated in [22].

**Adapting to alternative hardware connectivity.** In superconducting quantum hardware, two-qubit gates can be performed only between two qubits connected by a coupler. Increasing the connectivity is challenging due to crosstalk problems that arise as the number of couplers increases (and especially when they intersect). The surface code is already advantageous in this aspect, requiring only a square lattice with degree-4 connections and no long-range connectivity. However, it is notable that this requirement can still be relaxed, as [23] gives a method to implement the surface code on a “brickwork” lattice (a subset of the connections of a square lattice and topologically equivalent to a hexagonal lattice) with only degree-3 connections. This is achieved by regarding stabilizer measurement as a “stabilizer folding” procedure and realizing that one can change the direction of folding slightly in the middle of a round.

A coupler can also be defective, even if the qubits that it connects are not. Building upon the approach in [23,24] proposes a scheme that can efficiently handle both qubit and coupler defects.

### 6.2. Decoding

**Real-time decoding.** As we have described previously, the decoder is a classical algorithm that takes a decoding graph and detector values at each vertex as the input and outputs a set of edges as corrections, which is in turn used to adjust the results of logical measurements. The speed of the decoder is mainly important for *feedback control*, i.e., when the results of logical measurements determine what operations should be carried out next. It turns out that it is acceptable if there is a constant latency between the decoder receiving the detector values and outputting the decoding results, since in the worst case the lattice surgery circuit can go into “memory experiment mode” and wait for the decoder. However, if the average speed of the decoder cannot catch up to the hardware, the latency will keep increasing, causing an exponential backlog that may erase the advantage of quantum computation.

The real-time decoding problem is solved in [25,26,27] with a sliding-window parallelization scheme, where detector data are cut into overlapping windows and fed into multiple decoding units. The overlap allows each window to be decoded independently while the corrections in adjacent windows remain consistent.

**Improving accuracy beyond matching.** When we introduced the MWPM decoder in Section 4 and later extended it to lattice surgery, one detail we overlooked is that the decoding graph should be *weighted* to reflect the different probabilities of different types of error: An error with probability *p* should be associated with an edge with weight log((1−p)/p) . Then we can find the matching with the minimum total weight, instead of just the minimum number of edges, which should correspond to the most likely error configuration on the decoding graph.

However, such a decoding algorithm is still far from optimal. One problem is *degeneracy*: when there are many paths of equal or similar lengths between two vertices, and they all differ from each other by stabilizers, then the probabilities they represent should be added together, which may allow them to overpower another path that is individually slightly more likely but does not have that many alternatives.

Another bigger problem lies in the decomposition of all errors into *Z* and *X* errors. Such a decomposition would be accurate if, for example, Pr(Yerror)=Pr(Zerror∨Yerror)·Pr(Xerror∨Yerror) for any given qubit, so that after the decomposition, the *Z* error and the *X* error are independent events. However, this is generally not the case, and often we instead have, e.g., Pr(Yerror)=Pr(Xerror) , meaning that once we choose an edge on the *Z* decoding graph, there is no more cost associated with choosing the corresponding edge on the *X* decoding graph. To fix this problem, *Y* errors should instead be modeled as *hyperedges* connecting up to 4 vertices, but hypergraph matching is a much more difficult problem than ordinary graph matching.

Many works deal with this *Z*–*X* correlation problem in an ad hoc way by reweighting the decoding graph, beginning with [28], which simply runs the MWPM algorithm twice, reweighting the decoding graph with information obtained from the first run. This approach is quite crude but already leads to a big accuracy improvement. A further accuracy improvement is obtained in [29] by using the belief propagation (BP) algorithm to reweight the decoding graph. By introducing an aspect of randomization, Ref. [30] makes use of an ensemble of decoders to take into account different possible first-round matchings, with the advantage of being able to run many decoders in parallel for speedup.

However, currently the decoders with the best accuracy and reasonable efficiency are not matching variants but ones based on deep neural networks, with AlphaQubit [31] as a leading example. In addition to handling the *Z*–*X* correlation and degeneracy problems better, the neural network decoder is also able to adapt to error sources on the hardware that may not be well-modeled, such as crosstalk and leakage, through fine-tuning a network initially trained on simulated data. A main disadvantage that seems to be shared by all neural network decoders is the need for a large amount of initial training data, which apparently grows exponentially with the code distance *d*. Networks trained for small values of *d* generally do not generalize to larger *d*, and it is yet unclear whether new barriers might appear when scaling neural network decoders beyond d=11 .

### 6.3. Efficient Logical Operations

**Post-selection strategies.** At the end of Section 5.3, we have mentioned that it is usually better to declare failure when any error is detected by the Reed–Muller code than to try to correct it. Such a strategy is generally known as *post-selection*. Post-selection strategies go well with *resource states* such as the magic state, which can be generated repeatedly with relatively low cost. In contrast, post-selection is usually not very useful with intermediate states of long-running quantum circuits, since rejecting the state would require re-running the circuit from the beginning anyway.

Post-selection can be applied not only to distillation errors but also to topological errors (i.e., errors representable as strings in the defect diagram). This is particularly relevant during state injection, where short, undetectable error strings exist near the injection site. In particular, Ref. [32] demonstrates the effectiveness of post-selection when the fidelity of single-qubit gates is significantly better than that of two-qubit gates.

**Distillation strategies.** We have described the 15-to-1 distillation protocol above for its relative simplicity, but there are also many distillation protocols with other parameter combinations. For example, Ref. [12] also describes a 20-to-4 distillation protocol, but only has a fault-tolerant distance of 2, which means that post-selection is mandatory, and the output error rate is $O(p^{2})$ rather than $O(p^{3})$ . In general, Ref. [12] notes that it is necessary to carefully balance the output-to-input ratio, the output fidelity, and the rejection rate for post-selection.

Distillation can also involve non-stabilizer states other than T|+〉 . For example, Ref. [33] describes an efficient protocol for distilling 8 copies of T|+〉 into CCZ|+++〉 with fault-tolerant distance 2. The CCZ|+++〉 state can be directly used to implement a Toffoli gate, or it can be transformed back to two copies of T|+〉 with a catalyst T|+〉 state. This approach is especially effective for logical circuits with a large number of Toffoli gates.

When there are multiple levels of distillation, a simple optimization, used in [33], is to reduce the surface code distance for lower levels, since it is unnecessary to suppress topological errors to be much lower than injection/distillation errors at these levels.

**Magic state cultivation.** For a state injection protocol with only one non-stabilizer operation, the fidelity of the output state must be upper bounded by the fidelity of that operation. However, with additional non-stabilizer operations, it is possible to “cross-check” the injected state and increase its fidelity, as first proposed in [34], which made use of the color code and its transversal $H_{XY}$ operation. Developing on this idea, Ref. [35] introduces *magic state cultivation* by adding an aspect of growing the code distance over time.

The magic state cultivation scheme of [35] also starts in a color code, where all detected errors are post-selected away instead of corrected. The need for post-selection limits the distance that the code can grow to during this stage. Then, the code is rapidly expanded into a “grafted code”, at which point the decoder switches from post-selecting to correcting. Eventually, the code transitions to a matchable code that can be used directly in lattice surgery. Although magic state cultivation is not indefinitely scalable, under practical physical error rates, it can produce very good magic states, potentially suitable for direct use in large logical circuits, at only a fraction of the spacetime cost of distillation.

**Lattice surgery compilation.** As can be seen in some of the examples in Section 5.2, lattice surgery is a very flexible technique with a lot of freedom. Clifford gates can be either explicitly implemented using logical ancilla qubits or deferred to the end of the circuit, where it changes the bases of logical measurements, as demonstrated in [12]. Qubits can move in a spatial direction instead of the temporal direction, and some seemingly necessary wires in the circuit model can be eliminated through topological deformation of the defect diagram. As a profound example, Ref. [12] shows that the 15-to-1 distillation protocol can actually be implemented with only 5 long-term logical qubits, with 15 input magic states being introduced into the circuit one-by-one. The resulting circuit not only uses fewer qubits but also has a lower spacetime volume compared to a naive implementation.

For small modules that occur repeatedly in logical circuits, such as the distillation module, it may be possible to “hyper-optimize” them through an exhaustive search; Ref. [36] implements this approach with a classical SAT solver. For larger logical circuits, we may need to approach the problem similarly to classical compilation, possibly borrowing some techniques there. There exist some open-source compilers for lattice surgery, such as https://latticesurgery.com/ (accessed on 20 January 2026) and TQEC (https://tqec.github.io/tqec/index.html (accessed on 20 January 2026)), but, in general, there is still much research to be done in this area.

## Data Availability

No new data were created or analyzed in this study.
